# Age-related and postmenopausal breast cancer progression and treatment management: The significance of pro-inflammatory cytokines and CXC chemokines

**DOI:** 10.1016/j.gendis.2025.101606

**Published:** 2025-03-19

**Authors:** Amin Ullah, Rajeev K. Singla, Dan Cao, Boyang Chen, Bairong Shen

**Affiliations:** a Department of Abdominal Oncology, Cancer Center of West China Hospital, and Institutes for Systems Genetics, Frontiers Science Center for Disease-related Molecular Network, West China Hospital, Sichuan University, Chengdu, Sichuan 610212, China; b School of Pharmaceutical Sciences, Lovely Professional University, Phagwara, Punjab 144411, India; c School of Automation and Industrial Internet, Chongqing University of Posts and Teleconnections, Chongqing 400065, China

**Keywords:** Age, Breast cancer, CXC chemokines, Obesity, Postmenopausal women, Pro-inflammatory cytokines, Theranostic strategies

## Abstract

Older age is one of the leading risk indicators for advanced breast cancer. It is critical to extensively investigate how aging affects breast cancer, considering the increasing rate of population aging. Human body aging and death are caused by cellular senescence and alterations in the aging microenvironment *in vivo*. Breast cancer cells may invade more easily with age due to the stiff extracellular matrix of the breast. Furthermore, growing evidence suggests that the massive release of inflammatory immune mediators, such as cytokines (interleukins) or CXC chemokines (CXCs), and their receptors (CXCRs), including interleukin (IL)-6, IL-8/CXCL8, tumor necrosis factor (TNF), interferon (INF), transforming growth factor (TGF), CXCL1, CXCL9, CXCL10, CXCL11/CXCR3, and CXCL12/CXCR4, plays a critical role in the development of breast cancer in elderly patients. Researchers are particularly interested in obesity-induced inflammation because it has been shown to raise the risk of breast cancer in postmenopausal women with higher body mass index. Obesity-triggered inflammation causes increased infiltration of proinflammatory cytokines, adipokines, immune cells, and tumor cells in the enlarged adipose tissue of postmenopausal women with breast cancer, thereby modulating the tumor's immune-mediated microenvironment. Therefore, in this review, we focus on the functional significance studies of proinflammatory cytokines, CXCs, and CXCRs and describe their roles in influencing breast cancer progression in older women and their factors, such as obesity in postmenopausal women. In addition, the current status and prospects of cytokine- and CXC-based theranostic interventions for breast cancer therapy in elderly and postmenopausal women are discussed.

## Introduction

Breast cancer is the most common cancer to affect women, and it is a severe and life-threatening illness that is well-acknowledged as a significant cause of cancer mortality.^1^ As per the 2022 statistics of the American Cancer Society, breast cancer accounted for 11.6% of the total number of new cancer cases in 2022, with 2.3 million new cases diagnosed.^2^ A more in-depth knowledge of the molecular and cellular mechanisms that enable cancer cells not only to survive but also to proliferate is crucial, as breast cancer metastasis serves as a leading cause of mortality among women despite significant advancements in diagnosis and treatment.^3^

The incidence of breast cancer increases with age, particularly among postmenopausal women, and women aged 55 and older have a higher risk of developing the disease.^4^, ^5^, ^6^, ^7^ In addition, patients (65 years and older) account for approximately 45% of breast cancer diagnoses, with 19% of patients over 75.^8^ Thus, these studies indicated that aging enhances the susceptibility to age-related diseases, resulting in heterogeneous elderly individuals with substantial fitness and frailty variations.^9^ An increased risk of side effects from medication is present in elderly individuals. To prevent giving unneeded treatment to patients, healthcare providers should accurately identify individuals with a high risk of recurrence and select the most appropriate course of action for each patient.

Over one-third of women experience a postmenopausal state throughout their lifetimes. Menopause is the definitive cessation of menstruation because of ovarian activity depletion, which occurs after 12 months of amenorrhea.^10^ Hormonal changes are one of the most significant physiological effects of menopause. Estriol (E3), estrone (E1), and estriol (E2) are the three forms of estrogen; they are all aromatic molecules and C18 steroids.^11^ With respect to a woman's life cycle features, such as menopause, pregnancy, and reproductive age, every form serves a certain purpose.^12^ Throughout the menstrual cycle and before menopause, meningeal cells synthesize androstenedione, a metabolic precursor to testosterone and E1 in the ovaries and peripheral tissues.^13^ With an average level of total estrogen of 100–250 pg/mL, granulosa cells convert androstenedione to E1 through the aromatase activity of CYP 19. After that, E3 is converted to E2.^13^^,^^14^ On the other hand, postmenopausal women observe a dramatic reduction in circulating E2 levels to 10 pg/mL,^15^ indicating that these women experience an estrogen deficit for half of their lives.

Age-related variations in body composition are prevalent among most women, and this period nearly coincides with menopause.^16^ Obesity occurs in a sequence that includes the formation of adipose tissue, the expansion of older adipocytes, and the proliferation and differentiation of newer adipocytes.^17^ This increase in adipose tissue has multiple consequences for the hormonal balance of the body. Adipose tissue is a complex and highly active endocrine organ that participates significantly in many aspects of body function, such as nutrition regulation and energy storage.^18^ Obesity is primarily associated with increased visceral fat, which raises insulin hormone activity to a substantial degree and is one of the main causes of cancer. Menopause can accelerate the development of visceral fat because it lowers circulating estrogen levels, which can cause subcutaneous fat to decrease and visceral fat to increase. Estrogen has been associated with the buildup of subcutaneous fat.^19^ This change in fat distribution suggests that estrogen plays a major role in the development of postmenopausal obesity, which should be considered when addressing cancer formation. Furthermore, premenopausal women tend to accumulate adipose tissue in the gluteal femoral subcutaneous compartment, whereas postmenopausal women often have a larger total body fat mass, fat percentage, and central fat buildup.^20^ Variations in circulating endogenous sex hormone levels help to explain this in part because estrogen androgen receptors are found in both subcutaneous and visceral adipocytes.^21^^,^^22^ Sex hormones can act on their target cells because androgens attach to the androgen receptor, and estrogens bind to the estrogen receptor alpha/beta.^23^ Consequently, reducing the circulation of sex hormones will alter their function in target cells.

With these alterations in hormones, menopausal women are more vulnerable to numerous metabolic problems since considerable studies have been undertaken on the involvement of estrogen in various metabolisms, immunity, and inflammatory processes in animals and humans.^24^ Estrogens have been linked to inflammatory reactions in women. While it is well-recognized that estrogens exacerbate autoimmune disorders,^25^ reduced estrogen promotes susceptibility to infectious illnesses, as indicated by inadequate innate immune responses to viral infection in ovariectomized (OVX) mice.^26^ Moreover, OVX increased the susceptibility of female rats to dyslipidemia together with a reduction in innate cytokines, indicating compromised immunological and metabolic homeostatic responses due to estrogen depletion.^27^

The pathology of breast cancer is complex and involves several risks; however, postmenopausal obesity^4^^,^^28^^,^^29^ has drawn particular interest. Many studies have demonstrated that obesity increases the risk of developing breast cancer and increases the probability of death from the disease.^30^ Earlier research has linked body mass index (BMI) to a higher risk of breast cancer in postmenopausal women.^30^ Furthermore, abnormalities in the adipokine profile, including cytokines and C-X-C motif chemokines (CXCs), have a significant effect on the advancement of breast cancer^31^ because they stimulate the development and invasion of cancer and promote other tumor microenvironment (TME) cells to initiate invasion.^32^ The subsequent section provides an in-depth analysis of the precise molecular mechanisms by which immune parameters, including proinflammatory cytokines (interleukins) and CXCs, contribute to the progression of breast cancer with older age and risk factors, including obesity in the postmenopausal state, as determined by clinical and preclinical research.

## Pro-inflammatory cytokines and CXCs

Cytokines are small protein molecules released into the body and have a molecular weight of less than 40 kDa. They are generated by practically every cell in the body to control and impact the immune response.^33^ The secretion of pro-inflammatory cytokines will stimulate the production of further cytokines as well as the stimulation of immune cells.^34^ Thus, when the phrase "cytokine storm" first appeared, it defined inflammation as the unexpected up-regulation of an inflammatory process caused by the release of cytokines.^35^ Recent studies, however, suggest that any immune response must include the simultaneous release of pro- and anti-inflammatory cytokines.^36^ Cytokines are referred to by multiple names, including interleukins (ILs), chemokines, and growth factors.^37^ The so-called superfamilies that collectively make up cytokines rarely describe common genes but rather related structural features.^38^

**Figure 1:**
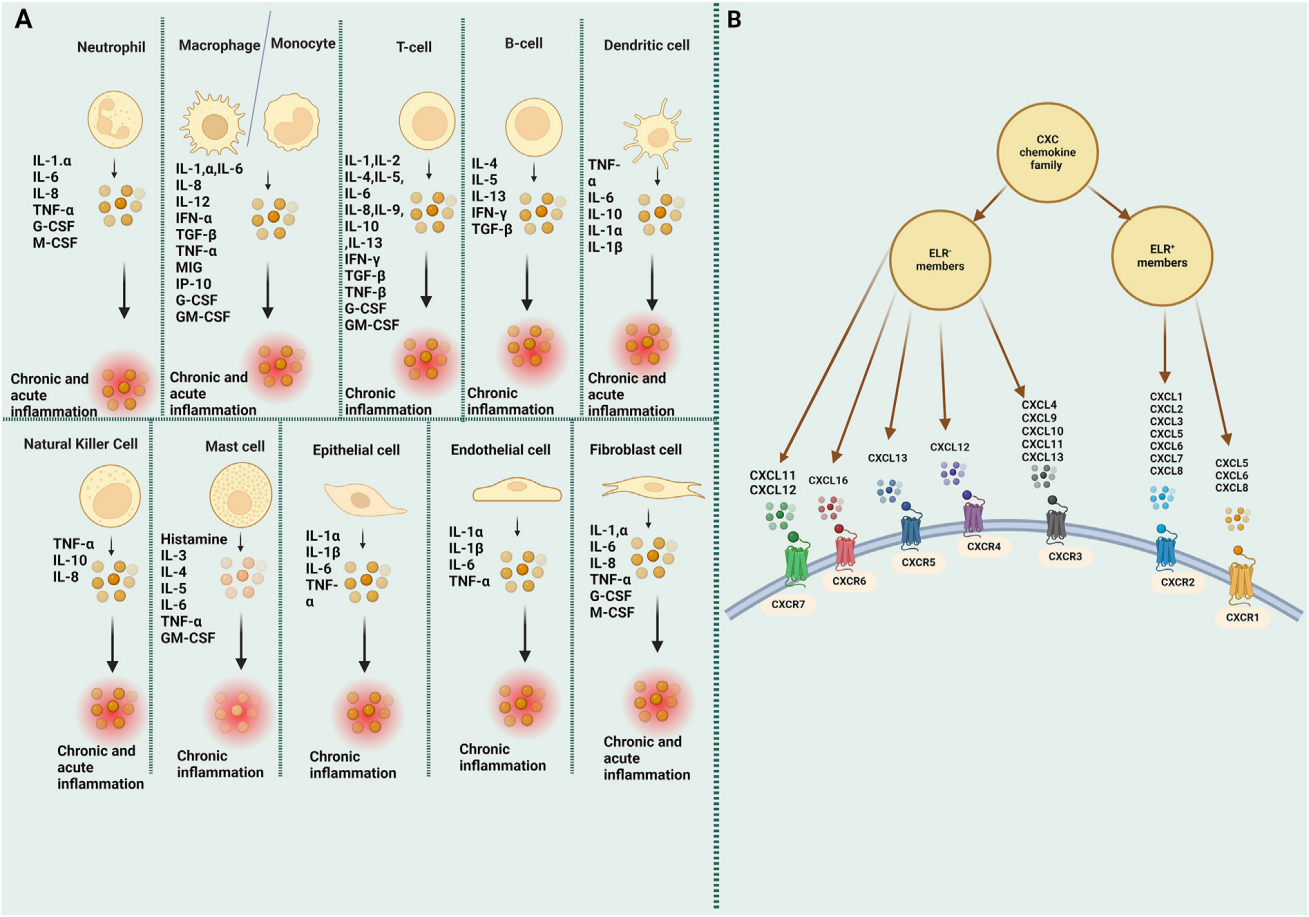
Pro-inflammatory cytokines and the C-X-C motif chemokine (CXC) family members and their receptors. **(A)** A diagram of various cells that act as major sources of pro-inflammatory cytokines, including interleukins (ILs), natural killer (NK), tumor necrosis factor-alpha (TNF-α), mast cells, transforming growth factor-beta (TGF-β), granulocyte-macrophage colony-stimulating factor (GM-CSF), interferon gamma-induced protein 10 (IP-10), granulocyte colony-stimulating factor (G-CSF), and monokine induced by gamma (MIG). **(B)** The classification of the CXC chemokine family.

Moreover, numerous cell groups can produce the same cytokine. Cytokines are pleiotropic because their actions vary depending on their target cell.^37^ Moreover, multiple cytokines may deliver similar outcomes, making them redundant. Their combined effects, however, might be synergistic. Eventually, they have the potential to trigger signaling cascades, which could result in disastrous results even with small amounts of protein.^39^ Most proinflammatory cytokines (ILs) are produced by Th1 cells, macrophages, and monocytes to exacerbate inflammation.^40^, ^41^, ^42^, ^43^ Figure 1A provides an overview of the secretion of inflammatory cytokines and their respective cells.

Small secretory proteins CXCs have four highly conserved cysteine amino acid residues and one non-conserved residue between the first two.^44^ CXC chemokine family members are classified as ELR^+^ or ELR^−^ based on the presence or absence of a Glu-Leu-Arg (ELR) motif in the NH2-terminus.^45^ ELR^−^ members include CXCL4/9/10/11/12/13/14 and CXCL16, while ELR^+^ members include CXCL1/2/3/5/6/7/8 and CXCL17.^46^^,^^47^ For CXCs, CXC chemokine receptors (CXCRs) are seven-transmembrane, G protein-coupled receptors. There are seven members in the CXCR family: CXCR1–CXCR7. The ligands for CXCR1 or CXCR2 are the ELR^+^ CXCs, whereas the ligands for CXCR3, CXCR4, CXCR5, CXCR6, or CXCR7 are predominantly the ELR-CXCs.^47^, ^48^, ^49^ The information regarding the classification of CXCs is provided in Figure 1B.

CXCs are the main chemokines in distribution and localization, and many of their genes have previously been defined.^50^ Their bigger serpentine G protein-coupled receptors (GPCRs) regulate the several roles of CXCs in organisms with multiple cells, including the distinctive cell movement.^51^ CXCs are "inflammatory" because they are released in response to inflammatory stimuli that trigger leukocyte recruitment to damaged or infected sites.^52^ We recently discussed the classification of CXCs and their effects on immune surveillance in several inflammatory diseases, such as diabetes, non-alcoholic fatty liver disease, liver cancer, endometriosis, and polycystic ovary syndrome. These CXCs function as chemotaxis, attracting immune cells and causing inflammation.^47^^,^^53^ We will thus have new opportunities for a better understanding of the role of cytokines and CXCs in the genesis of inflammatory disorders if we have a more profound knowledge of their potential mechanisms in inflammatory disorders and their management.

## Molecular subtypes of breast cancer and cytokine network

Breast cancer is heterogeneous and characterized by a number of molecular and morphological subtypes.^54^ Invasive breast carcinoma of no special type (IBC NST) is the most prevalent morphological type. Its molecular subtypes are distinguished by three primary phenotypes: luminal (estrogen receptor- and/or progesterone receptor-positive), human epidermal growth factor receptor 2 (HER2)-overexpressing, and triple-negative.^55^ The metastatic potential of breast cancer cells is correlated with the molecular subtype: luminal tumor subtypes exhibit a reduced malignant progression rate and a more favorable response to therapy than the HER2-overexpressing and triple-negative subtypes of breast cancer.^56^ Moreover, recent studies revealed that cytokines are crucial for the advancement and development of breast cancer molecular subtypes.^57^ The most specific cytokines for the luminal BHER2-positive molecular subtype are IL-6 and IL-8/CXCL8. Because IL-6 and IL-8 potentiate each other's biological effects and alter the activity of steroidogenesis enzymes, they can have a direct or indirect impact on the expression of estrogen, progesterone, and HER2 receptors by tumor cells in IBC NST of the luminal B HER2-positive subtype.^58^, ^59^, ^60^, ^61^

**Figure 2:**
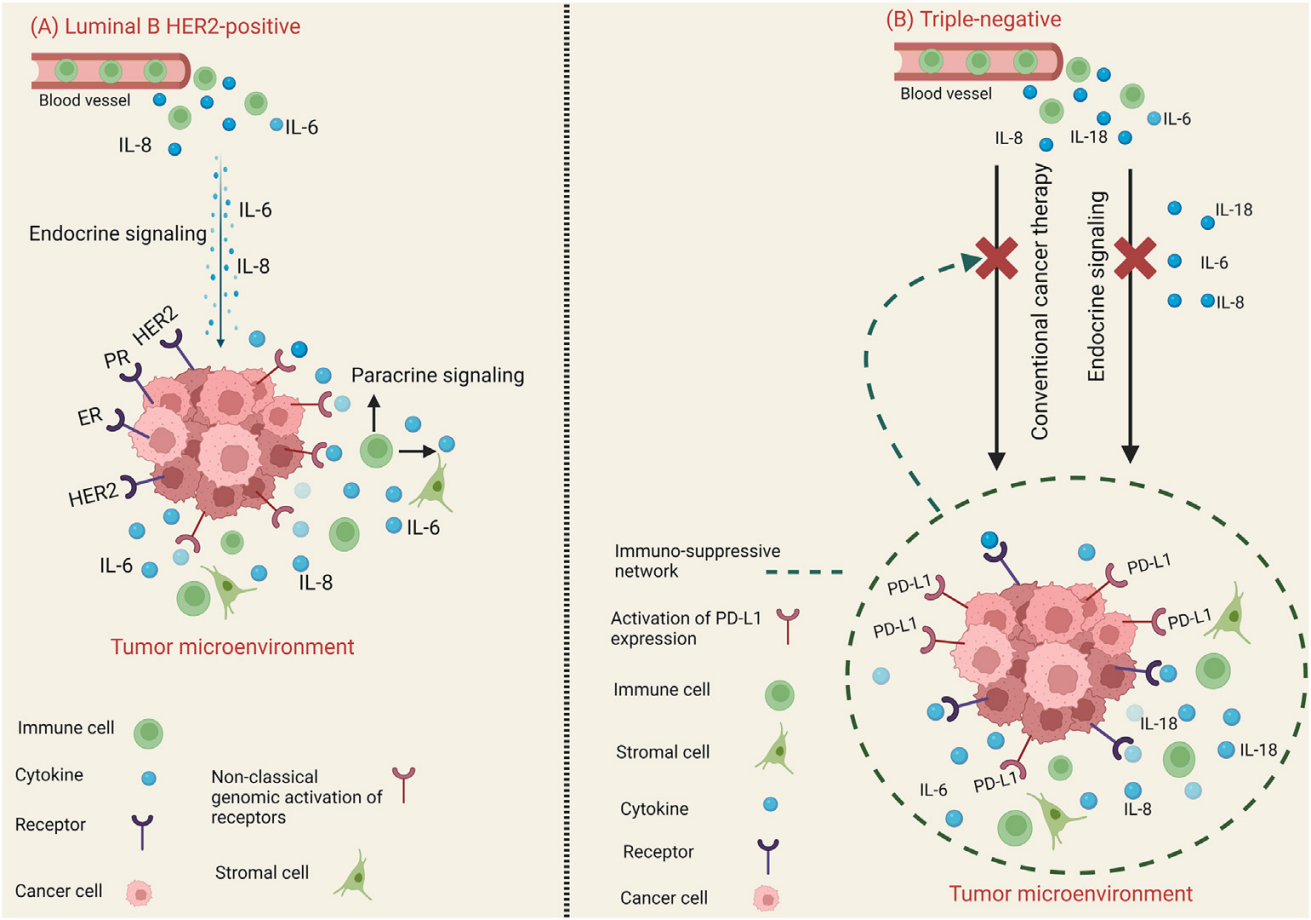
The cytokine network in various molecular subtypes of breast cancer. **(A)** The cytokine network in luminal B HER2-positive subtype. **(B)** The cytokine network in triple-negative subtype.

Furthermore, the overexpression of HER2 triggers the HER2-IL-6- signal transducer and activator of transcription 3 (STAT3) signaling cascade, which stimulates breast cancer stem cells to self-renew and develop resistance to anti-HER2 therapy.^62^^,^^63^ Based on the published data, the high spontaneous secretion of IL-6 and IL-8 by cultured tumors of the luminal B HER2-positive subtype is likely the result of the activity of the cells that comprise the tumor, including its microenvironment. It is also possible that these cytokines can contribute to the high expression of estrogen and progesterone receptors and the HER2 receptor, which defines the luminal B HER2-positive subtype (Fig. 2A).^64^ Furthermore, by assessing the cytokine-producing capacity of IBC NST, specific features of the triple-negative molecular subtype, such as the lowest polyclonal activator stimulation index (SIPA) for IL-6 and IL-8 secretion and lower SIPA for IL-18 secretion, can be determined in comparison to luminal subtypes. The observed variations suggest that polyclonal activators (PA) have a suppressive effect on cytokine production by IBC NST of this molecular subtype. It is plausible that in IBC NST of the triple-negative molecular subtype, IL-6, IL-8, and IL-18 collectively constitute an immunosuppressive network (Fig. 2B)^64^ since IL-6, IL-8, and IL-18 are known to decrease immune responses in tumor microenvironment cells and to promote the development of multidrug resistance.^65^, ^66^, ^67^

In patients with breast cancer that have the luminal B HER2-positive molecular subtype, an anti-cytokine therapy based on IL-6 and IL-8 receptor inhibitors may be useful. The research indicates that in a model of breast cancer, IL-6 receptor inhibitors prevent bone metastases.^68^ An anti-cytokine treatment involving IL-6, IL-8, and IL-18 receptor inhibitors is likely to suppress the intra-tumoral immunosuppressive network regarding the triple-negative molecular subtype of breast cancer. This will likely occur through nonspecific suppression of programmed cell death-ligand 1 (PD-L1) expression, among other mechanisms.^65^

A recent study by Autenshlyus and others found that spontaneous and mitogen-induced cytokine production by cultured tumors and peripheral blood cells from IBC NST patients is linked to breast cancer molecular subgroups. The luminal B HER2-positive molecular subtype exhibits the highest spontaneous and mitogen-induced secretion of IL-6, IL-8, IL-1Ra, and TNF-α in cultured tumors, as well as the highest secretion of IL-2, IL-6, IL-8, IL-1β, TNF-α, interferon-gamma (IFN-γ), and granulocyte colony-stimulating factor (G-CSF) in cultured peripheral blood cells.^64^ Based on their interaction with estrogen and progesterone receptors and receptor HER2, these cytokines may modulate the synthesis of enzymes involved in estrogen and progesterone metabolism. The triple-negative subtype of IBC NST has the lowest cytokine-producing capability of cultured tumors for IL-6 and IL-8 and lower SIPA values for IL-18 secretion than luminal subtypes. Further, XYZ (luminal A, luminal B HER2-negative, and HRE-2 enriched) is linked to a suppressive effect of the tested set of mitogens (PA) on the secretion of cytokines by triple-negative molecular subtype-cultured tumors.^64^ In IBC NST of the triple-negative molecular subtype, the cytokines generated by the cultured tumors (IL-6, IL-8, and IL-18) likely combine to form an intra-tumoral immunosuppressive network (Fig. 2B).^64^ Consequently, these results suggest that the cytokine network exhibits unique functional characteristics in IBC NST of luminal B HER2-positive and triple-negative molecular subtypes.

## Menopause and aging impact breast cancer biomarker profiles

Our most recent study revealed that chemokines and cytokines are crucial for elderly cancer patients.^69^ As patients age, the prevalence of breast cancer rises. According to estimates, postmenopausal women account for 80% of breast cancer incidence, with half of cases diagnosed between 50 and 69.^70^ In addition, recent research has shown that postmenopausal women with breast cancer have an up-regulation of the inflammatory cytokine IL-6, suggesting that these cytokines are crucial for the inflammatory process in such a disease condition.^71^^,^^72^ When 164 breast cancer patient samples were tested when they were menopausal, researchers discovered a favorable link between the expression of TNF-α and IL-6 and breast cancer cells.^73^ Interestingly, anti-cytotoxic T-lymphocyte antigen 4 (CTLA4) and anti-PD-L1 responses in older mice are significantly lower than in younger mice in preclinical studies of triple-negative breast cancer (TNBC).^74^ Further, compared with older mice, younger animals showed an enhanced gene signature for IFN signaling and antigen presentation. The authors discovered that individuals under 40 years old had higher levels of IFN and inflammatory signaling than patients over 65 years old, which is consistent with the preclinical evidence.^74^ Research suggests that elevated levels of pro-inflammatory cytokines may contribute to an increased risk of breast cancer by delaying the age-related atrophy of the mammary lobes.^75^ Furthermore, there has been increasing concern about the impact of age on tumor-infiltrating lymphocytes (TILs). The research investigated TILs in patients with luminal B (ER^+^PR^+^HER2^−^) breast cancer who were 35–45 years old, 55–65 years old, and older (>70 years old).^76^ Further, TILs undergo immunohistochemical phenotyping utilizing cluster of differentiation (CD)3, CD4, CD5, CD8, CD20, CD68, and forkhead box protein P3 (FOXP3). The findings indicated that a decrease in the overall percentage of stromal TILs in biopsies was related to age (*P* = 0.025). Furthermore, aging significantly impacted the immune infiltrate/tumor composition, with a substantial decrease in the density of specific immune cells detected by CD3, CD5, CD8, and CD20 in all tumor areas (*P* < 0.042). In each site of the tumor, the percentages of CD8^+^ TILs also dramatically declined with age (*P* < 0.0001). Age did not, however, affect the patterns of TIL distribution throughout any tumor area. The authors also noted that there were notable aging-related alterations in the plasma concentrations of a number of inflammatory mediators, including IL-1a, interferon-gamma-induced protein 10 (IP-10), and IL-8 ^76^. According to these findings, innate immune activation with age and immunological dysfunction due to aging are both likely to have failed. Table 1 provides a comprehensive overview of the evidence on comparisons regarding the expression levels of immune modulators (cytokines and CXCs) between pre- and postmenopausal, elderly, and young breast cancer studies in the clinical and preclinical setting. Moreover, studies have demonstrated that breast cancer patients in the postmenopausal phase express high levels of CXCL1, CXCL3, CXCL12/CXCR4, and CXCL8 and that these levels also increase the aggressiveness of the tumor environment. These CXCs may account for some tumor types' aging-related increase in aggressiveness.^77^^,^^78^ These studies indicate that cytokines and CXCs affect the progression of postmenopausal women's inflammation and breast cancer. Moreover, the clinical and preclinical studies of breast cancer and their age with menopausal status are displayed in Table 2.

**Table 1:** Comparative analysis of cytokine and CXC chemokine expression in age-related, pre-, and post-menopausal breast cancer: insights from preclinical and clinical studies. Symbols ↑ indicated high concentration, ↓ low concentration, and ↔ no change.

Disease	Target	Expression level in pre-menopausal/young	Expression level in post-menopausal/old	Study type	Sample	Main role	References
Breast cancer	IL-6	≤40 years ↓	≥65 years ↑	Clinical	MCF-7 cells	Inflammation	^142^
	IFN	≤12 weeks mice, human, ≤40 years ↑	≥65 years, and mice >12 months ↓	Clinical/preclinical	Blood and tumor tissue	An age-related decline in IFN signaling in murine and human triple-negative breast cancer tumors predicted reduced anti-PD1 and anti-CTLA4 efficacy	^74^
	IL-8/CXCL8, TNF-α, IL-1 and IL-4	≤6 months mice ↓	>20 months mice ↑	Preclinical	ECM/MDA-KTB21 and MB-23 cells	Inflammation	^220^
	IL-6/IL-6R and TNF	≤34 years ↓	≥65 years ↑	Clinical	Blood	IL-6, IL-6R, TNF, or TNFRSF1A contribute to the risk of breast cancer in aged patients	^221^
	IL-7, IL-22, TGF-β, TNF-α and CXCL9	<60 years ↓	≥60 years ↑	Clinical	Blood	The up-regulation of these cytokines and chemokines plays a role in depression and fatigue in older breast cancer patients	^222^
	IL-1Ra and IL-8	≤50 years ↓	>50 years ↑	Clinical	Blood	These cytokines act as tumor immunoregulation in older patients	^223^
	IL1RN	≤35 years ↓	≥55 years ↑	Clinical	Tumor tissue	Inflammation	^224^
	IL-1α, IL-8, CXCL10	≤45 years ↓	≥70 years ↑	Clinical	Blood and tumor tissue	Plasma inflammatory cytokines (IL-1a) and chemokines (CXCL10, IL-8) all induce chronic inflammation in aged breast cancer patients	^76^
	IL-6 and TNF-α	Pre-menopausal ↑	Post-menopausal ↑	Clinical	Blood	Correlated with the development of breast cancer	^225^
	IL-6	Pre-menopausal ↓	Post-menopausal ↑	Clinical	Blood and tumor tissue	Inflammation	^189^
	IL-6 and TNF-α	Pre-menopausal ↓	Late post-menopausal ↑	Clinical	Blood	Inflammation and alteration in bone marrow microenvironment for breast cancer recurrence	^226^
	IL-6, TNF-α, IL-8, and TGF-β	IL-6, TNF-α, and IL-8 were associated with a higher percent mammographic density (PMD) among premenopausal women	Higher expression levels (above median) of the anti-inflammatory marker TGF-β were associated with lower PMD among postmenopausal women	Clinical	Tumor tissue	Influence breast carcinogenesis through their effects on the PMD	^227^
	IL-8Rβ and TGFβ2	Pre-menopausal ↑	Post-menopausal ↓	Clinical	Tumor tissue	Angiogenesis, cancer cell growth and survival	^228^
	IL-8, IL-10 and TNF	For pre-menopausal women, significant associations with breast cancer risk were observed for IL-8 TT genotype, IL-10 TT genotype, and TNF c.-418 GA and AA genotypes.	For post-menopausal women, significant associations with breast cancer risk were observed for IL-8 TT genotype, IL-10 TT genotype, TNF c.- 418 GA and AA genotypes, and TNF c.-488 GA genotype	Clinical	Blood	Increased risk of breast cancer	^229^
	IL-1B rs1143627	Pre-menopausal ↔	IL-1B rs1143627 “A” allele in post-menopausal	Clinical	Blood	IL-1B rs1143627 “A” allele plays a protective role in the development of breast cancer among postmenopausal women	^230^
	TGFβ1	Pre-menopausal ↑	Post-menopausal ↓	Clinical	Tumor tissue	High TGFβ levels were shown to be generally associated with poor prognosis in breast cancer	^231^

**Table 2:** The clinical and preclinical breast cancer studies according to age and menopausal status. Age is the mean age. The ↑ symbol indicates upregulation of proinflammatory cytokines and CXC chemokines.

Age/years	**Menopausal Status**	Target	Study type	Expression	Target tissue	Main role	References
78		CXCL10	Clinical	↑	Blood	Risk factors of breast cancer	^160^
65	Postmenopausal	IL-6 and TNF-α	Clinical	↑	Blood	Inflammation	^246^
55	Postmenopausal	CXCL10	Clinical	↑	Breast cancer tissue/	Resistance to chemotherapy	^203^
78	Postmenopausal	IL-6	Clinical	↑	Blood	Inflammation	^113^
70		IL-8	Clinical	↑	Breast cancer Tissue/Blood	Recruit tumor-associated macrophages	^238^
79	Postmenopausal	IL-6	Clinical	↑	Blood	Inflammation	^72^
≥50	Postmenopausal	IL-1, IL-6, and TNF-α	Clinical	↑	Mammary tissue	Inflammation	^177^
≥50	Postmenopausal	CXCL12/CXCR4	Clinical	↑	Breast cancer tissue	Biomarker predicting lymph node metastasis	^78^
52	Postmenopausal	IL-6, IL-1β and IL-17	Clinical	↑	Blood	Inflammation	^214^
52 and > 60	**Menopausal**	IL-6 TNF-α and KDM2A	Clinical	↑	Tumor tissues	Fibroblast senescence	^110^
54	Postmenopausal	Nuclear factor erythroid 2–related factor 2 (NRF2) and IL-11	Clinical	↑	Tumor tissues	NRF2-driven tumorigenesis	^124^
55	Postmenopausal	IL-6 and TNF-α	Clinical	↑	Blood	Inflammation	^217^
59	Menopause	CXCL9/10/11	Clinical	↑	Breast cancer tissue	Macrophage marker and immunosuppression-related genes	^159^
60	Postmenopausal	IL-6	Clinical	↑	Blood	Inflammation	^112^
90		IL-6/10/TNF-α	Clinical	↑	Blood	Executive function	^115^
<70	Postmenopausal	IL-6, TNF-α, IL-8 and IL-10	Clinical	↑	Breast cancer tissue	Inflammation	^75^
≤70	Postmenopausal	CXCL1/3/8	Clinical/preclinical	↑	Blood	Shorter relapse-free survival	^77^
72		IL-8 and IL-6	Clinical	↑	Blood	Inflammation	^109^
≥80	Menopausal	Fibroblast growth factor 13 (FGF13) and IL-8	Clinical	↑	Breast cancer tissue	Senescence-associated secretory profile	^260^
58	Postmenopausal	IL-6 and TNF-α	Clinical	↑	Breast adipose tissue	Increase the adipocyte size and macrophage number	^73^
69	Postmenopausal	IL-6 and TNF-α	Clinical	↑	Blood	Inflammation of adipose tissue	^71^
73	Postmenopausal	IL-1β, IL-6 and TNF-α	Clinical	↑	Blood	Low-grade inflammation and increase the risk of postmenopausal breast cancer	^178^
94	Postmenopausal	IL-1 and IL-8	Clinical	↑	Blood	Decreased insulin level	^182^
50	Postmenopausal	IL-RA and IL-12	Clinical	↑	Blood	Fatigue	^183^
79	Postmenopausal	IL-6	Clinical	↑	Fat tissue	Inflammation	^174^
85	Postmenopausal	TNF-α, IL-6	Clinical	↑	Blood	Negative correlation to Omentin-1, which indicated that reduced the omentin-1 activity	^186^
65	Postmenopausal	IL-6	Clinical	↑	Blood	Promotes tumor progression and metastasis	^187^
85		IL-10	Clinical	↑	Tumor tissues	IL-10 can suppress T-cell proliferation and activity in breast cancer	^122^
>65		IL-11	Clinical	↑	Tumor tissues	IL-11Rα positive correlated with the expression of tumor cells and bone metastasis	^126^
73		IL-11	Clinical	↑	Tumor tissues and blood	IL-11 is associated with bone metastasis	^125^
74	menopause	IL-6	Clinical	↑	Adipose tissue and blood	Inflammation	^189^
50	Postmenopausal	IL-10	Clinical	↑	Adipose tissue	IL10 increased the recruitment of IL10-producing T regulatory cells by the obese adipose, which influences premenopausal breast cancer risk	^196^
53	Postmenopausal	TGF-β, IL6, TNF-γ and IFN-γ	Clinical	↑	Blood and adipose tissue	WAT inflammation	^199^
62	Postmenopausal	TNF-α and IL-6	Clinical	↑	Blood	Correlated to resistin and induce inflammation	^202^
79 and > 60		CXCL9/CXCL10	Clinical	↑	Breast cancer tissue	Poor prognostic characteristics	^162^^,^^163^
10 weeks	Diet-induced obesity mice in postmenopausal	IL-6	Preclinical	↑	Adipose tissue	Impaired NK cell receptors	^184^

Aging is marked by an ongoing decrease in physiological integrity, which results in impaired performance. This degradation is the leading cause of major human pathologies such as cancer, cardiovascular disease, neurodegenerative conditions, and hyperglycemia.^79^^,^^80^ Growing evidence suggests that the risk of developing cancer increases with aging and is associated with poor prognosis. Various aging-related changes, such as immune system dysregulation, could explain this trend. Patients over 65 are diagnosed with approximately half of all malignancies.^81^^,^^82^ In addition, senescent cells increase with age.^83^ They show a senescence-associated secretory phenotype (SASP), which means they release cytokines or CXCs or inflammatory immune mediators, such as CXCL1 and CXCL8/IL-8, that may help tumors grow by creating a tumorigenic environment.^84^, ^85^, ^86^ Eventually, as people age, their immune systems deteriorate, making it challenging to create an effective immune response against developing cancers.^87^

Moreover, as we mentioned, several pro-inflammatory cytokines and CXCs act as immunological mediators between aging and reproductive cancers. However, none have made it from the bench to the bedside.^88^, ^89^, ^90^, ^91^, ^92^ The existing literature reveals a solid connection between these immune signaling molecules and breast cancer advancement in the respective population. Therefore, we will focus on the association between breast cancer in older/postmenopausal individuals and their risk factor (obesity), as well as the role of inflammatory immune mediators, including cytokines and CXCs, in this review study. These inflammatory cytokines and CXCs in older/postmenopausal individuals may be promising predictors of breast cancer progression. These molecules may also be targeted for treatment to improve outcomes for this high-risk population.

## Inflammatory cytokines and aging-associated breast cancer

### IL-1

The IL-1 family of cytokines initiates and regulates inflammatory and immunological responses. The eleven-member IL-1 family includes pro- and anti-inflammatory cytokines and receptor antagonists.^93^ IL-1α and IL-1β, two IL-1 family members, share similar biological activities and attach to the type 1 IL-1 receptor (IL-1R1).^93^ Furthermore, the SASPs (CXCL1α/β, IL-1α/β, CXCL8/IL-8, and IL-6) are crucial regulators of chronic inflammation and aging phenotypes, and they accumulate during healthy aging and in diseases associated with aging.^94^^,^^95^

**Figure 3:**
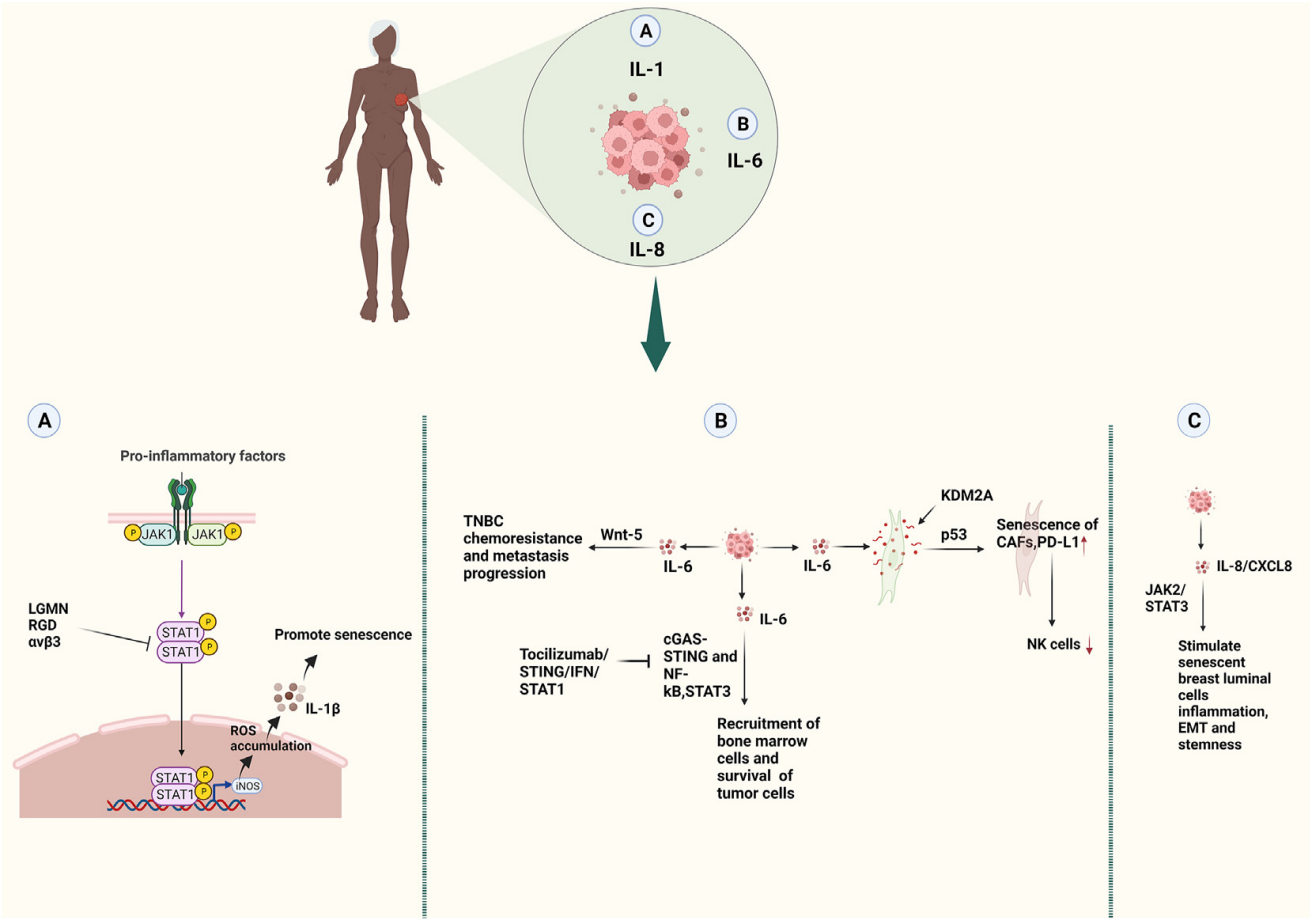
Pro-inflammatory cytokines (interleukins/ILs) play a role in aged/postmenopausal breast cancer women or animal model/senescence cells, as depicted in the illustration. **(A)** Schematic diagram of legumain (Lgmn^−/−^ mice) deletion mediating the inhibition of tumor-associated macrophage (TAM) pro-tumor effects and promoting tumor senescence. TAM-derived legumain suppresses signal transducer and activator of transcription 1 (STAT1) signaling in an autocrine manner. Deletion of legumain led to the sustained activation of STAT1 signaling, stimulating *inducible nitric oxide synthase (*iNOS) expression and intracellular reactive oxygen species (ROS) accumulation, and thus contributing to the increased expression and secretion of IL-1β and promoting tumor senescence. The regulatory effect of legumain secretion on STAT1 inhibition via interaction with integrin αvβ3 in TAMs. **(B)** In aged patients, IL-6 is up-regulated, which recruits bone marrow cells and plays a role in the survival of tumor cells. These processes can be inhibited by tocilizumab/STING/interferon (IFN)/STAT1 pathways. IL-6 can stimulate the expression of lysine-specific demethylase 2A (KDM2A) in normal fibroblasts, transforming them into cancer-associated fibroblasts (CAFs). Up-regulation of KDM2A in these cells induces senescence in fibroblasts, which is dependent on p53, and enhances the release of IL-6. Inducing senescence in CAFs up-regulates programmed cell death-ligand 1 (PD-L1), which reduces the natural killer (NK) cells to target the tumor microenvironment (TME). The up-regulation of IL-6 induces chemoresistance and metastasis progression in triple-negative breast cancer (TNBC) through the Wnt-5 pathway. **(C)** In both *in vitro* and an animal model of breast cancer, the up-regulation of IL-8/C-X-C motif chemokine ligand 8 (CXCL8) induces senescent of breast cancer, inflammation, epithelial–mesenchymal transition (EMT), and stemness via the Janus kinase 2 (JAK2) and STAT3 pathways. The red color up arrow symbol (↑) represents up-regulation, while the down arrow symbol (↓) represents down-regulation.

In invasive breast cancer, the proteolytic enzyme legumain controls cell growth. Older breast cancer patients have high legumain levels.^96^ Before we begin to explain the action of legumain in tumor-associated macrophages (TAMs), we must first understand that there are two central TAM polarization states in the TME: pro-inflammatory "M1" and pro-tumor "M2". Research on the role of legumain in TAMs revealed that legumain involvement in TME polarization acts as legumain depletion in TAMs that leads to persistent activation of the Janus kinase 1 (JAK1)/signal transducer and activator of transcription 1 (STAT1) signaling pathway, which was postulated to control the M1 phenotype. Following that, M1 induces cellular senescence, which has a tumor-inhibitory impact. The molecular mechanism of the inhibitory action is the activation of the STAT1/*inducible nitric oxide synthase* (iNOS)/reactive oxygen species (ROS) axis; M1 secretes IL-1β, leading to breast cancer senescence (Fig. 3A).^97^ On the other hand, it can be concluded that legumain, when bound to integrin αvβ3, may facilitate the M2 polarization of wild-type TAMs while preventing the M1 polarization. Therefore, TME's legumain can be used for therapeutic purposes to decrease tumor immune escape.

IL-1A is a systemic aging biomarker that rises with age and becomes a key player in the aging process.^98^ Based on a search, IL-1A levels were more significant in breast cancer susceptibility gene 1 (BRCA1) carriers than in the comparable controls.^99^ This suggests that the IL-1A gene contributes to breast cancer in older women.

### IL-2

The master activation factor IL-2, which mediates pro- and anti-inflammatory immune responses and promotes the proliferation and differentiation of natural killer (NK) and helper/regulatory T cells, is also linked to bone metastases in breast cancer. The anti-tumor immune response cytokine IL-2, which is released by T-helper leucocytes and has no predictive value, was found to be present in higher concentrations in therapy responders, according to the findings of a paper by Iakovou et al that there were evaluated levels of serum cytokines in women with bone metastases receiving radionuclide palliative therapy (RTP).^100^ A previous long-term pilot study^101^ showed that immunotherapy, namely the cyclic administration of IL-2, considerably increased the survival time of postmenopausal patients with endocrine-dependent metastatic breast cancer. In addition, an assessment of the cellular immunity in these patients revealed that only a minor increase in eosinophils occurred during the disease. Further evidence that sIL-2Ra may suppress an endogenous anti-tumor immune response originates from the correlation between higher blood concentrations of soluble IL-2 receptors (sIL-2Ra) and fewer TILs in the tumors in breast cancer patients.^102^ Prognostically favorable variables, including ER-positive tumors and older age, are correlated with increased NK cell amounts, β2-microglobulin, or sIL-2Ra,^102^ which age supports the concept that the immune system has a role in the progression of early breast cancer in older individuals.

In contrast, total lymphocytes, CD4^+^, CD8^+^, and CD16^+^56^+^ cells increased significantly following IL-2 administration during clinical benefit.^101^^,^^103^ These findings imply that IL-2 immunotherapy is a significant aspect of cancer treatment for patients. In addition, it has been demonstrated recently that gamma delta (γδ) T cells exhibit promise anti-tumor efficacy in the context of cell-based immunotherapy.^104^ The study found that T-cell receptor-independent activation of γδ T cells with cytokines, including IL-2, IL-12, and IL-18, increased their anti-tumor potential. γδ T cells promote apoptosis by cytotoxic substances such as granzymes and perforin, as well as tumor cell (MCF7) senescence via IFN-γ and TNF production.^104^ In several clinical trials, IL-2 has been shown to target numerous types of tumor-associated antigens, including CD20, epithelial cellular adhesion molecules, and carcinoembryonic antigen (CEA).^105^ IL-2's pharmacokinetic profile can also be changed by combining it with antibodies that bring the cytokine to the TME of breast, lung, colon, and stomach cancers or binding covalently to polyethylene glycol molecules and other molecules. The IL-2 variants are linked to a particular antibody in cergutuzumab amunaleukin (CEA-IL2V, RG7813) to target CEA *in vivo*,^106^ and it is being tested with atezolizumab (NCT02350673). In phase I/II, RO7284755, an IL-2 variant immunocytokine connected with an anti-programmed cell death (anti-PD1) moiety, is tested for safety and anti-cancer efficacy (NCT04303858).

### IL-6

Breast cancer cells can migrate and invade *in vitro* when exposed to SASPs, particularly those that significantly generate IL-6 from the senescent MDA-MB-231 breast cancer cell line and the senescent MCF-10A regular epithelial cell line.^107^ It was discovered that MCF-7 breast cancer cell production of vimentin, SNAIL-2/SlugZEB-1, and SNAIL-1 was increased by IL-6 in *senescence-conditioned medium* from senescent foreskin fibroblasts, hydroxycarboxylic acid receptor 2 (HCA2). These alterations indicate that MCF-7 cells are undergoing epithelial–mesenchymal transition (EMT), which provides cancer cells with a greater capacity for tumorigenesis.^108^ Moreover, IL-6 can stimulate the proliferation of MCF-7 cells, and it is interesting to note that this occurs due to a particular IL-6/IL-8 ratio rather than a rise in cytokines.^109^

Chen et al^110^ recently reported that lysine-specific demethylase 2A (KDM2A) expression can be stimulated in normal fibroblasts by cancer-derived cytokines, including IL-6; this results in the transformation of these fibroblasts into cancer-associated fibroblasts (CAFs). The increased expression of KDM2A in these cells promotes p53-dependent senescence in fibroblasts and increases IL-6 secretion. In turn, it promotes the proliferation of cancer cells. Moreover, an increase in PD-L1 expression is induced by elevated levels of KDM2A in CAFs; this mechanism subsequently decreases the recruitment of NK cells to target the TME (Fig. 3B). It provides an immunosuppressive TME for breast cancer progression.

Recent research has shown that elderly breast cancer patients and animal models have higher levels of IL-6, which are linked to frailty and the tumor progression process.^91^^,^^111^^,^^112^ Additionally, IL-6 levels were higher in older survivors with higher levels of lipopolysaccharide-binding protein (LBP), sCD14, and LBP/sCD14 than in younger survivors with higher gut permeability biomarkers. Intestinal permeability is particularly pro-inflammatory in older breast cancer survivors.^113^ Furthermore, recent research suggests that the stimulator of interferon gene (STING)–IFN–STAT1 signaling pathway generally induces apoptosis in TNBC cells. On the other hand, chromosome (CIN) instability promotes tumor cell viability by inducing IL-6-STAT3-mediated signaling via the cGAS-STING and nuclear factor-kappa B (NF-kB) pathways.^114^ Further, IL-6 inhibits STING–IFN–STAT1 signaling when activated by NF-kB signaling.^114^ Clinical blockage of IL-6 signaling by the IL-6 receptor-targeting drug tocilizumab may hinder IL-6-STAT3 signaling and slow the growth of tumors. Consequently, this stimulates NF-kB-related signaling, the release of specific cytokines such as IL-6, and the activation of IL-6-STAT3 signaling to promote the recruitment of bone marrow cells and the survival of tumor cells (Fig. 3B). Likewise, a recent study found that elderly breast cancer survivors scored inferiorly long-term neurocognitively than controls; higher IL-6 levels partially explained this association.^115^

The latest study identifies a hybrid cell population in human breast cancer enriched in senescent cells, and these senescent cells secrete SASPs such as IL-6, which follow WNT-5 pathways and contribute to TNBC chemoresistance and metastatic progression (Fig. 3B).^116^

### IL-8/CXCL8

Bergen et al^76^ recently conducted a comprehensive immune biomarker study on patients of different ages diagnosed with luminal B-like breast malignancies to gain valuable insights. The blood immunological profile and the local immune response exhibited many significant age-related changes, multiple of which may be associated with immunosenescence and/or inflammation. Aging was associated with substantial alterations in the plasma concentrations of various inflammatory mediators, including IL-8 and IL-1α. These results confirmed that patients with luminal B-like breast cancer undergo age-related and frailty-related remodeling of both systemic immunity and the tumor immune response. It suggests that tumor and inflammatory mediators interact differently with aging.

In addition, IL-8-dependent, senescence-related active fibroblasts help primary human luminal cells change into multipotent stem cells.^117^ The dedifferentiation of luminal cells elicited by IL-8 is mediated by miR-141 and STAT3-dependent p16INK4A down-regulation.^118^ Moreover, increasing evidence indicates that IL-8 is a central regulator of breast cancer stem cell activity and plays a crucial role in EMT.^119^ The dedifferentiation of luminal cells into multipotent stem cells, which may be IL-8-dependent, raises the possibility of proliferative mammary stem cells accumulating in response to inflammation, stress, and age. Thus, mammary stem cells that produce IL-8 may be precious for cosmetic and therapeutic purposes and a valuable tool for drug development and biological research.^118^

Furthermore, senescent luminal cells stimulated stromal fibroblasts via the Janus kinase 2 (JAK2)/signal transducer and activator of transcription 3 (STAT3) pathway in an IL-8-dependent manner. Based on the results from *in vitro* and an animal disease model, these myofibroblasts accelerated EMT and stemness processes in breast cancer cells. These findings indicate that senescent breast luminal cells stimulate fibroblasts in an IL-8-dependent manner, contributing to the inflammatory/carcinogenic microenvironment (Fig. 3C).^117^

Likewise, it has been suggested that the extra centrosome-associated secretory pathway (ECASP), which induces the release of several pro-invasive and SASP factors (such as growth differentiation factor 15 (GDF15) and IL-8), which are linked to tumorigenesis and tumor metastasis, may be responsible for these notable alterations.^120^ These results support the idea that cells with extra centrosomes may be a source of pro-tumorigenic substances, including IL-8, suggesting that possessing more centrosomes may be advantageous for tumors. Thus, the development of novel precision medicine strategies aimed at centrosome-phagy may be crucial for the effective use of anti-tumor therapeutics.

### IL-10 and IL-11

**Figure 4:**
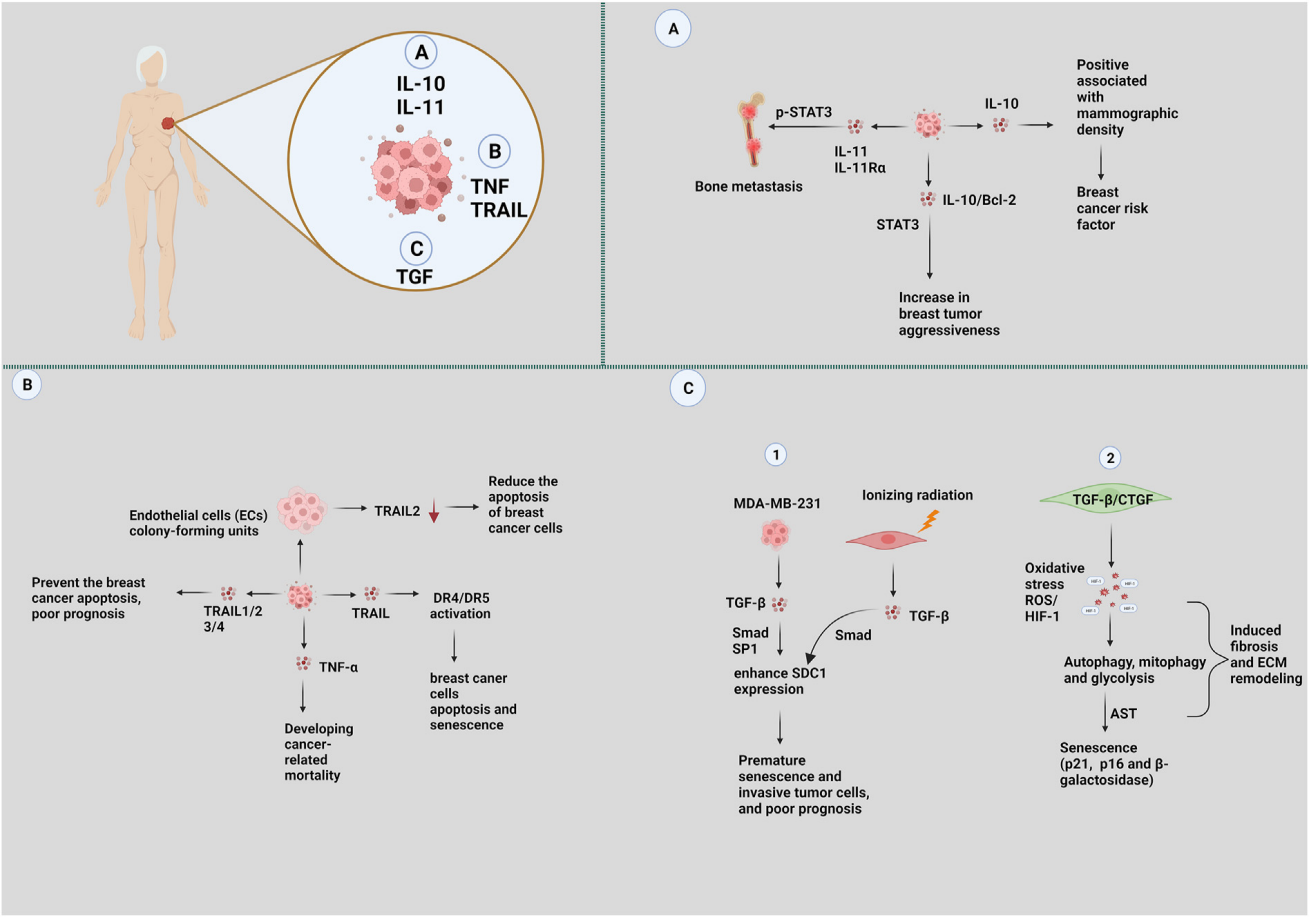
Interleukin (IL)-10/11/tumor necrosis factor (TNF) and transforming growth factor (TGF) pro-inflammatory cytokines play a role in breast cancer in postmenopausal women and senescence cells, as shown in the illustration. **(A)** There is a correlation between higher expression of B-cell lymphoma 2 (Bcl-2) family proteins and IL-10, and they contribute to the aggressiveness of breast cancer via signal transducer and activator of transcription 3 (STAT3) pathway. The significant activation of IL-10 positive is correlated with mammographic density during breast cancer. Aged patients secrete a large amount of IL-11 and receptor IL-11Rα, which contribute to bone metastasis through the p-STAT3 pathway. **(B)** Higher levels of the inflammatory marker TNF-α are strongly associated with an increased risk of cancer-related mortality in elderly patients. TNF receptors, such as TNF-related apoptosis-inducing ligand (TRAIL) 1, 2, 3, and 4, play a crucial role in preventing cancer cell apoptosis and progression. However, TRAIL activation of death receptor 4/5 (DR4/5) promotes apoptosis in breast cancer cells but may also induce senescence. Endothelial cells (ECs) down-regulate the expression of TRAIL2, which reduces the apoptosis process in breast cancer. **(C1)** The breast cancer cell line MDA-MB-231 enhances surface proteoglycan syndecan 1 (SDC1) expression in young and senescent breast fibroblasts via TGF-β in a paracrine manner. On the other hand, exposure to ionizing radiation in early passage human breast stromal fibroblasts can cause premature senescence. This results in the formation of an autocrine TGF-β loop that leads to SDC1 overexpression via the Smad (small mothers against decapentaplegic) pathway. This suggests that ionizing radiation and invasive cancer cells have a synergistic effect leading to the overexpression of SDC1, which is a known poor prognostic factor in breast cancer development. **(C2)** TGF-β/connective tissue growth factor (CTGF) expression in stromal fibroblasts leads to reactive oxygen species (ROS) production, resulting in the stabilization of hypoxia-inducible factor 1 (HIF1)-transcription factor. HIF1-activation then promotes the induction of autophagy, mitophagy, and glycolysis. Autophagy drives the onset of senescence via the autophagy–senescence transition (AST), resulting in the up-regulation of cyclin-dependent kinase (CDK) inhibitors (p21 and p16 as well as β-galactosidase) (a lysosomal enzyme and marker of senescence). Oxidative stress, autophagy, and senescence may also contribute to CTGF-induced fibrosis and extracellular matrix (ECM) remodeling. The symbol of a red arrow pointing downwards (↓) is used to indicate down-regulation.

Hanna et al examined the inflammatory profile of age-related lobular involution and noted that chronic inflammation may decrease completion and raise breast cancer risk. Further, lobular involution was negatively linked to IL-6, TNF-α, and IL-8 levels, while mammographic density,^75^ a known breast cancer risk factor, was positively linked to IL-10 levels (Fig. 4A).^121^ The unexpected inverse connection with lobular involution may be due to its complex and controversial role in breast cancer development. Llanes-Fernándeza et al^122^ previously reported that 85% (23 out of 27) of the breast cancer tissue studied from elderly individuals showed significant IL-10 expression (STAT3 in cancer cells). IL-10 showed significant correlations with apoptosis markers. IL-10 showed a positive correlation with B-cell lymphoma 2 (Bcl-2) and Bcl-2 associated X-protein (Bax) and an inverse correlation with p53. 89% of p53-negative individuals had IL-10 expression. Researchers suggest that the presence of IL-10 and increased expression of Bcl-2 family proteins in the TME indicate an increase in the aggressiveness of breast tumors (Fig. 4A).^122^ As expected, it was demonstrated that IL-10 production was increased in breast cancer cells.^123^

Nuclear factor erythroid 2-related factor 2 (NRF2) is abnormally activated due to the dysregulation of the Kelch-like ECH-associated protein 1 (KEAP1)-NRF2 system, which was initially discovered as a crucial defensive mechanism against oxidative stress. Patients with cancer, notably those with lung and breast cancer, have a significantly poorer prognosis when there is an increased formation of NRF2 in their tumors.^124^ Furthermore, breast cancer patients have substantially elevated NRF2 and IL-11 levels. NRF2-dependent carcinogenesis enhanced IL-11 expression. These data show that NRF2 and a microenvironment signal trigger IL-11. This study shows how the microenvironment impacts cancer cell NRF2 pathways and secretory phenotypes.^124^ In the NRF2 addiction cancer model, IL-11 disruption significantly suppressed carcinogenesis, implying that IL-11 plays an essential role in NRF2-driven tumorigenesis.^124^ Thus, this study implies that IL-11 may be a promising therapeutic target for breast cancers linked to NRF2.

IL-11 is essential in the osteolytic cycle, interacting with cancer and bone cells. Researchers have proposed that IL-11 is an osteolytic factor in human breast cancer cells. Breast cancer cells expressing high levels of IL-11 also exhibit higher rates of bone metastases.^125^ Evidence suggests that IL-11, via STAT3 phosphorylation, contributes to bone metastasis in patients with breast cancer-related bone metastasis (Fig. 4A).^125^ Furthermore, in patients with advanced breast cancer, there was a reported positive connection between the expression of IL-11Rα in tumor cells and the incidence of bone metastases.^126^ IL-11/IL-11Rα is connected to bone metastasis and could potentially predict bone metastases from breast cancer.

### TNF

Chronic inflammation is linked to processes that can cause cancer to develop or proliferate. A study examined the associations between circulating levels of the inflammatory markers IL-6, *C-reactive protein* (CRP), and TNF-α with overall cancer incidence as well as the incidence of cancers specific to certain sites (breast and prostate).^127^ Increased levels of inflammatory markers are more strongly linked to the chance of developing cancer-related mortality than cancer incidence (Fig. 4B). The authors have shown that the connections between IL-6, CRP, and TNF-α and cancer risk may be site-specific.^127^

TNF-related apoptosis-inducing ligand (TRAIL) is a recently discovered cytokine member of the TNF family.^128^ Several normal cells produce TRAIL, a cytokine that stimulates death receptor 4/death receptor 5 (DR4/DR5) to induce exogenous apoptosis in cancer cells. In MDA-MB-231 cancer cells exposed to doxorubicin or ionizing radiation, DR5 was overexpressed. Caspase-dependent death was promoted in senescent MDA-MB-231 cancer cells *in vitro* by the DR5-selective TRAIL D269H/E195R (DHER) while causing no harm to normal cells (Fig. 4B).^129^ DR5 receptor targeting may be an emerging, low-toxicity cancer therapeutic approach. In addition, Heilmann et al^130^ reported that approximately 25% of the breast cancer specimens analyzed exhibited heterogeneous expression of TRAIL receptors (R1, R2, and R4), which caused terrible prognoses for patients (Fig. 4B). Furthermore, the expression of TRAIL-R3 and -R4 inhibits the apoptotic process of breast cancer cells..^131^ According to the claims above, conducting more in-depth research on TRAIL receptor trafficking would enhance our understanding of how TRAIL receptor signaling is controlled and its role in cancer.

Endothelial cell colony-forming units (EC-CFUs) produced by breast cancer patients show decreased expression of genes associated with tumor necrosis factor receptor-2 (TNFR2) signaling, leading to resistance against TNF-α-mediated apoptosis (Fig. 4B).^132^ Evidence suggests that TNF-α, through its interaction with TNFR2 and autocrine stimulation of monocyte chemoattractant protein-1 (MCP-1), may facilitate myeloma cell movement across endothelial cells.^133^ Based on these results, medicines that target TNFR2 show great potential for addressing breast cancer.

### TGF

Bone morphogenetic protein 7 (BMP7), a member of the TGF-β family, promotes regulation of the telomerase reverse transcriptase (TERT) gene in a bone morphogenetic protein receptor II (BMP RII) receptor- and small mothers against decapentaplegic homolog 3 (Smad3)-dependent manner.^134^ A mutation in the BMP RII receptor prevents BMP7-induced TERT repression, which leads to longer telomeres, more significant telomerase activity, and sustained cell proliferation. Chronic exposure to BMP7 causes telomere shortening, replicative senescence, and apoptosis.^134^ TERT repression is necessary for the replication senescence and apoptosis caused by BMP7, as TERT overexpression can reverse BMP7-induced cell senescence.^134^ These results suggest that TGF-β-induced pulmonary senescence and pathological fibrogenesis depend on telomere shortening and TERT gene suppression through Smad3. In addition, scholars have postulated a correlation between increased levels of cell-surface proteoglycan syndecan 1 (SDC1) expression and the autocrine activity of TGF-β via the Smad pathway and the transcription factor SP1. This interaction may potentially contribute to the development of tumors and the promotion of angiogenesis (Fig. 4C1).^135^

Furthermore, previous investigations have shown that stromal cells lacking caveolin-1 (Cav-1) activate TGF-β signaling and enhance connective tissue growth factor (CTGF) transcription.^136^, ^137^, ^138^ CTGF induces the autophagy–senescence transition in CAFs.^136^ CTGF expression in stromal fibroblasts produces ROS, which regulates hypoxia-inducible factor 1 (HIF1). The subsequent promotion of autophagy, mitophagy, and glycolysis by HIF1 activation, the autophagy–senescence transition which is set off by autophagy, causes the overexpression of cyclin-dependent kinase (CDK) inhibitors (p21 (WAF1/CIP1) and p16 (INK4A)) and galactosidase, which is a lysosomal enzyme and a sign of aging. Oxidative stress, autophagy, and senescence may also be involved in CTGF-driven extracellular matrix remodeling (Fig. 4C2).^136^

A recent bioinformatic study discovered that TGFBI was a high-risk signature associated with the prognosis of breast cancer patients, with the high-risk group having a worse prognosis than the low-risk group.^139^ It has been found by Ruiz et al^140^ that bisphenol A has an impact on the TME in aged female mammary glands during neoplasia. Bisphenol A causes an increase in TGF-β1 and p-STAT3 in mammary cells, leading to EMT and inflammation. M1 and M2 macrophages establish the TME, while mast cells promote inflammatory chemokine signaling and communication for bisphenol A-disrupted epithelial cell EMT. The inflammation and signaling from bisphenol A can lead to a TME in elderly female mammary glands, which promotes tumor growth and invasion.

### IFN

Previous bioinformatic studies demonstrated that interferon signaling (TS-T1) genes, such as interferon-induced proteins with tetratricopeptide repeats 2 (IFIT2), IFIT1, IFITM1, and IFIH1, are enriched for higher expression in breast cancer patients.^141^ They also demonstrated that interferon signaling was the most enriched canonical pathway. Additionally, increased recurrence-free survival was linked to the up-regulation of TGFBI and interferon-induced proteins IFIT2, IFIH1, and IFI44L in the residual tumor. Shorter recurrence-free survival was associated with increased interferon signaling during chemotherapy in nonresponding patients, and it was hypothesized that this might be an immunological tolerance response in aggressive diseases.^141^ This study also implies that therapy drives alterations in interferon pathway signaling. It might be crucial for determining breast cancer recurrence risk factors and could suggest the potential benefits of immune-altering therapy in patients who do not respond to treatment.

Interestingly, the exceptionally long lifespans of centenarians are likely because of their ability to repair DNA damage and their resistance to inflammation effectively. Indeed, cells isolated from centenarians displayed a distinct anti-inflammatory molecular structure and minimal levels of DNA damage. Centenarians' dermal fibroblasts showed less pro-inflammatory factor expression than older adults, including low IL-6 and type 1 IFN-β cytokine levels and specific microRNAs linked to age-related inflammatory disorders.^142^

Interferon response pathways were found to be some of the most repressed pathways in myelocytomatosis oncogene (MYC)-activated malignancies. They examined the IFN-γ, IFN-α, and IFN-γ pathways characteristic of TNBC tumors; a high MYC signature was highly correlated with decreased interferon signaling in TCGA.^143^ In addition, MYC directly suppresses STING/IFN signaling in tumor cells with genomic instability. Genomic instability increases STING/IFN signaling. However, MYC-mediated suppression of the downstream effectors stops the immune system from responding, which stops immune cells from being recruited.^144^ These findings demonstrate that MYC inhibits innate immunity and promotes tumor immune evasion, which is responsible for the poor immunogenicity of MYC-overexpressing TNBCs. Likewise, it was noted that the interferon signature and PD-L1 expression were closely related, suggesting that PD-L1 expression represents the anti-cancer immune responses in breast cancer.^145^

Furthermore, interferons released by activated lymphocytes boost the production of PD-1 ligands. At the same time, granulocyte-macrophage colony-stimulating factor (GM-CSF) secreted by active lymphocytes triggers the overexpression of PD-1 ligands on macrophages via STAT3-related signals. Although IFN-γ is associated with PD-L1 overexpression, it has been proposed that GM-CSF produced from TILs can synergistically cause PD-L1 overexpression with IFN-γ via STAT3 signal activation,^146^ which suggests that the inflammatory microenvironment could induce the overexpression of PD-L1. Moreover, a recent study found that breast cancer patients classified in the immune-activated subclass had higher levels of IFN-γ, and it was projected that these patients would respond better to anti-PD-1 immunotherapy. These results show that the IFN-dominant and ER subtypes are linked to high immunogenicity, which points to immune class and activation features.^147^

High polymeric immunoglobulin receptor (PIGR) expression in breast cancer is associated with an improved 5-year survival rate. IFN-γ and IL-1β both boosted the expression of PIGR in breast cancer cells. Increased PIGR expression *in vivo* in breast cancer could reflect tumor-associated immune cells' polarized condition.^148^

**Figure 5:**
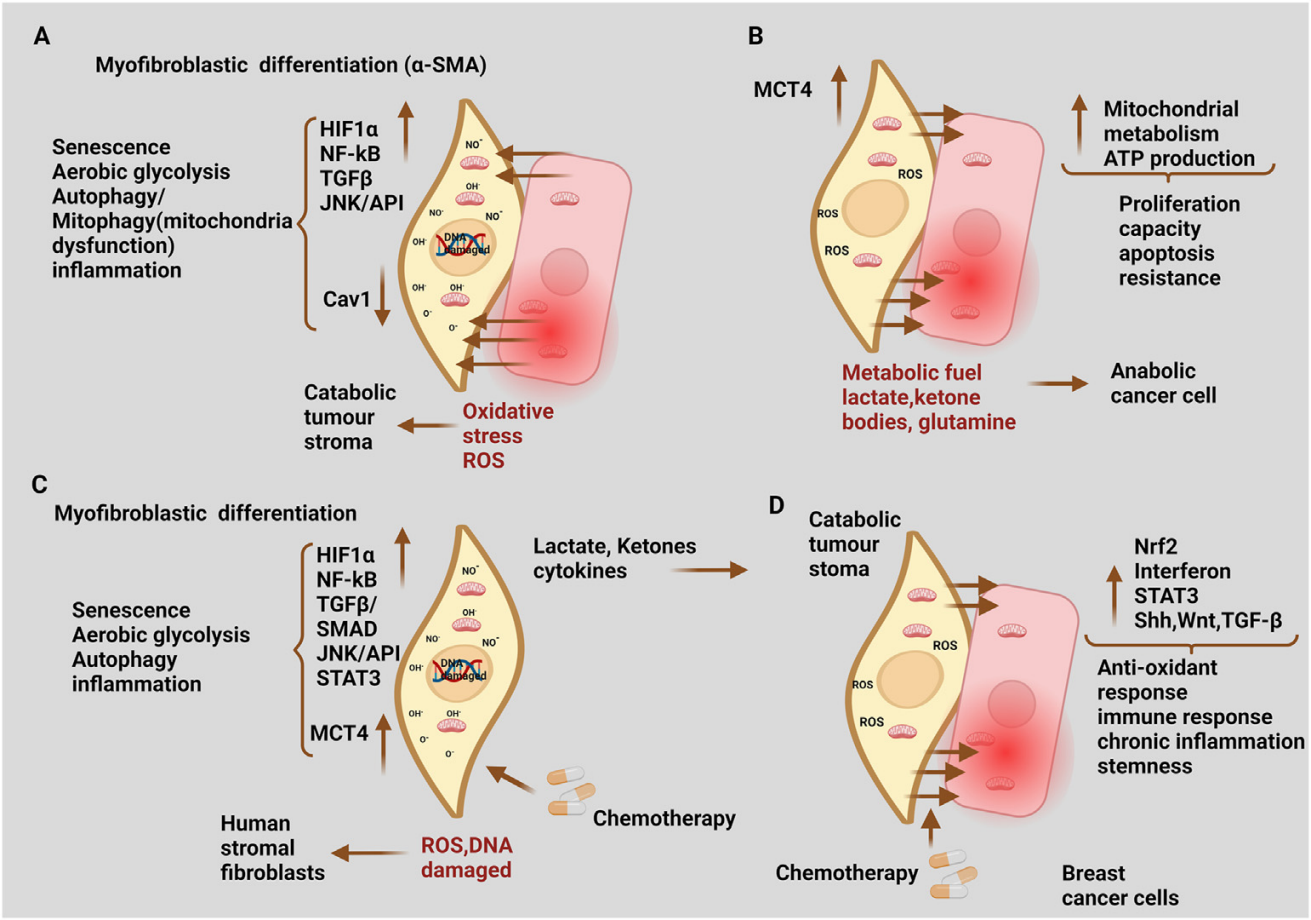
The stroma phenotype of catabolic tumor. **(A)** Reactive oxygen species (ROS) production by rapidly proliferating cancer cells causes oxidative stress in surrounding stromal cells, causing changes such as cancer-associated fibroblast (CAF) transformation, activation of hypoxia-inducible factor 1 (HIF1), nuclear factor-kappa B (NF-kB), transforming growth factor (TGF), or c-Jun N- terminal kinase (JNK)/activator protein 1 (AP1) signaling pathways, a switch to aerobic glycolysis and mitochondrial dysfunction, autophagy and senescence, and cytokine release: the catabolic tumor stroma phenotype. **(B)** Increased glycolysis and autophagy result in increased synthesis of energy-rich metabolites, such as lactate, which are produced by stromal cells and taken up by cancer cells, where they drive mitochondrial metabolism and adenosine triphosphate (ATP) production. Chemotherapy promotes the antioxidant response, immunological response, and stemness in cancer cells by inducing the catabolic tumor stroma phenotype. **(C)** Chemotherapy-induced DNA damage activates the HIF1, NF-kB, TGF, signal transducer and activator of transcription 3 (STAT3), and JNK/AP1 signaling pathways in stromal cells, promoting CAF differentiation, a switch to aerobic glycolysis and mitochondrial dysfunction, autophagy and senescence, the release of inflammatory cytokines, and the inhibition of interferon-mediated signaling. **(D)** When cancer cells encounter these catabolic fibroblasts, they respond to chemotherapy by activating antioxidant and immune response signals, as well as stemness. The red color up arrow symbol (↑) represents up-regulation, while the down arrow symbol (↓) represents down-regulation.

Chemotherapeutic drugs also have various effects on the CAF component of the TME, and these effects frequently have protumor effects. They can induce fibroblast senescence.^149^ Chemotherapeutic drugs may change fibroblasts into a CAF-like senescent phenotype that produces protumor inflammatory cytokines, such as interferons and IL-6, even when no surrounding tumor cells exist. The details are shown in Figure 5.^149^

### CXCs

Recent discussions have focused on the classification of CXCs and how they affect immune surveillance in various inflammatory diseases, such as cancer, diabetes, nonalcoholic fatty liver disease, endometriosis, and polycystic ovarian syndrome. These CXCs are chemotactic, recruiting immune cells and triggering inflammation. Our prior studies have demonstrated the importance of CXCs in different inflammatory diseases.^47^^,^^53^^,^^150^, ^151^, ^152^ Therefore, CXCs play a crucial role in the inflammatory process and are essential in identifying target therapeutics for genes associated with different inflammatory diseases, such as breast cancer, particularly in the older population.

### CXCL1

**Figure 6:**
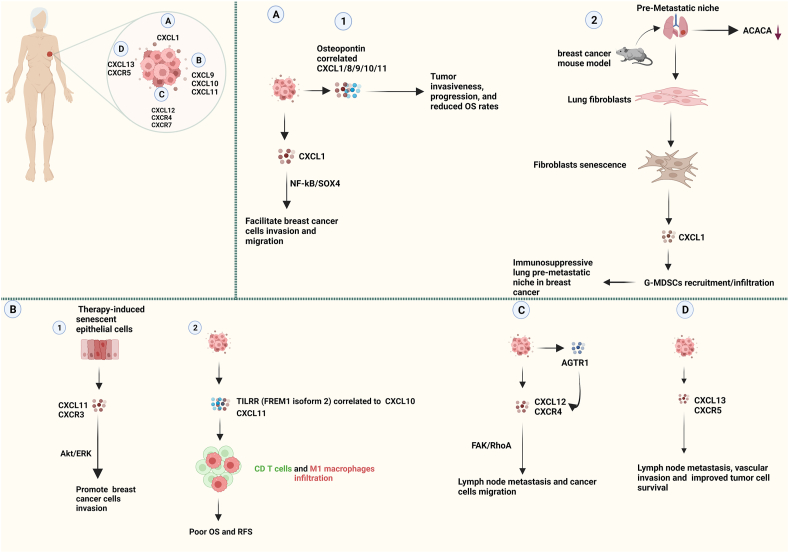
Pro-inflammatory C-X-C motif chemokines (CXCs) play a crucial role in the progression and development of breast cancer in aged patients, postmenopausal women, animal models, and senescent cells. **(A1)** The overexpression of CXCL1 leads to invasion and migration in breast cancer cells through the nuclear factor-kappa B (NF-kB)/SRY-box transcription factor 4 (SOX4) pathway. On the other hand, a high number of CXC chemokines are associated with the expression of osteopontin (OPN), which promotes tumor invasiveness and progression and reduces the overall survival (OS) rate. **(A2)** In the breast cancer mouse model, the primary breast cancer alters the lipid metabolism of lung fibroblasts by acetyl-CoA carboxylase alpha (ACACA) down-regulation. Lung fibroblasts convert to a senescent phenotype, which increases the recruitment of granulocyte myeloid-derived suppressor cells (G-MDSCs) by CXCL1 production, which induces an inflammatory lung microenvironment. **(B1)** Therapy-induced senescent epithelial cells can secrete senescence-associated secretory phenotype (SASP) factors that promote the invasion of breast cancer cells through the CXCL11/CXCR3/Ak strain transforming (AKT)/extracellular signal-regulated kinase (ERK) pathway. **(B2)** In older breast cancer patients, the secretion of TILRR (FREM1 isoform 2) is positively associated with the presence of immune cells like CD4^+^ and CD8^+^ T cells, CD86^+^ M1 macrophages, and the expression levels of CXCL10 and CXCL11. These factors are believed to be indicative of poor OS and recurrence-free survival (RFS). **(C)** The angiotensin II type I receptor (AGTR1) signaling regulates breast cancer migration and lymph node metastasis through the CXCL12/CXCR4 pathway. AGTR1 increases the level of CXCL12 in the lymph node, which attracts tumors that highly express CXCR4 cells. The mechanism behind AGTR1-induced migration and invasion of tumor cells involves up-regulating CXC12/CXCR4, Via focal adhesion kinase (FAK), and Ras homolog gene family member A (RhoA) molecules. **(D)** An aged breast cancer patient secretes a significant amount of CXCL13/CXCR5, which leads to lymph node metastasis, vascular invasion, and increased breast cancer cell survival.

The most common chemokine secreted by tumor-associated macrophages, CXCL1, is found on chromosome 4. In cancer, elevated CXCL1 levels have been associated with tumor cell invasion and development.^153^ Likewise, in breast cancer, CXCL1 may facilitate breast cancer invasion and migration via the NF-kB/SRY-box transcription factor 4 (SOX4) signaling pathway (Fig. 6A1).^154^ In addition, a bioinformatic analysis also discovered that the risk model genes were expressed in all TCGA cancers. CXCL1 expression was differentially elevated in older breast cancer patients.^155^

Osteopontin (OPN) is secreted by various cell types, including cancer cells, and plays a role in cell proliferation, angiogenesis, fibrosis, invasion, and metastasis.^156^ According to a recent study, the expression of OPN is higher in menopausal women with breast cancer. The study also found a correlation between the expression of OPN and the number of inflammatory CXCs, such as CXCL1/8/9/10/11 (Fig. 6A1).^157^ These inflammatory mediators are associated with tumor invasiveness, disease progression, and reduced overall survival rate.

Furthermore, metabolic reprogramming is a critical factor not only in the adjacent tissues of cancer but also in the formation of the pre-metastatic niche during breast cancer metastasis. According to Huang and colleagues,^158^ acetyl-CoA carboxylase α (ACCA) expression in lung fibroblasts is down-regulated by breast cancer, which modifies lipid metabolism and causes inflammation and fibroblast senescence. Furthermore, the SASP of lung fibroblasts may elevate CXCL1 secretion, which in turn stimulates the recruitment of immunosuppressive granulocyte myeloid-derived suppressor cells to establish an immunosuppressive lung pre-metastatic niche in breast cancer (Fig. 6A2).^158^

### CXCL9/CXCL10 and CXCL11

CXCL9, CXCL10, and CXCL11 have tumor-promoting abilities and have been associated with advanced human cancer, particularly in elderly breast cancer patients' progression and metastasis.^159^^,^^160^ According to Chen et al, a high level of expression of CXCL9/CXCR3 was associated with an improved overall survival rate in breast invasive carcinoma.^161^ Furthermore, it has been demonstrated that increased CXCL9 levels are linked to the density of TILs and are a reliable predictive biomarker for the TNBC subgroup.^162^ Overexpression of CXCL9 and CXCL10 in the luminal A subgroup of elderly patients was associated with a poor prognosis.^162^^,^^163^ Future assessments should consider the link between CXCs and breast cancer subtypes and outcomes. In the future, researchers should assess these data points.

A recent study has reported that senescent epithelial cells induced by therapy can secrete SASP factors, which facilitate the invasion of breast cancer cells via the CXCL11/CXCR3/Ak strain transforming (AKT)/extracellular signal-regulated kinase (ERK) pathway.^164^ Thus, targeting the CXCL11/CXCR3 axis could assist in reducing the adverse effects on the TME of therapy-induced senescent endothelial cells (Fig. 6B1).

Researchers recently discovered that as atherosclerosis progresses, TILRR (FREM1 isoform 2) up-regulates the expression of proinflammatory genes.^165^ Lower expression of FREM1 is frequently linked to TNBC and hormone-receptor-negative breast cancer. A strong correlation exists between the reduction and poor overall survival and recurrence-free survival. Furthermore, CD4^+^ and CD8^+^ T cell immunological infiltration, CD86^+^ M1 macrophages, and CXCL10 and CXCL11 expression levels are favorably connected with FREM1 expression. According to these results, FREM1 may be a valuable biomarker for determining and evaluating the level of immune infiltration and breast cancer prognosis (Fig. 6B2).^166^

### CXCL12/CXCR4/CXCR7 and CXCL13/CXCR5

Recent studies have linked the CXCL12-CXCR4 axis and human and animal breast cancer model development.^92^^,^^167^ Tumor development, angiogenesis, inflammation, and the immune system are all significantly influenced by angiotensin II type I receptor (AGTR1).^168^ Ma et al^169^ suggested that ATII receptor 1 (AT1R) plays a role in lymph node (LN) metastasis. Via focal adhesion kinase (FAK)/Ras homolog gene family member A (RhoA) signaling promotes cell migration and LN metastases. Whereas CXCL12/CXCR4 signaling assists tumor cells in reaching LNs, FAK/RhoA signaling controls cell migration. These findings were validated using the renin-angiotensin system, which modifies drugs to prevent cancer and its progression (Fig. 6C).

The breast TME may alter because of radiation's ability to cause tumor stroma to become prematurely senescent. Increasing metastasis and recurrence may be a result of these alterations. A study attributes the radiated tumor bed effect or accelerated growth of breast tumors following radiation to the molecule CXCL12/CXCR4.^170^ Senescence is a primary contributor to the radiated tumor bed effect via SASP, as demonstrated by the combined results of these findings and the effectiveness of senotherapeutics in inhibiting radiation-induced breast tumor growth.

In addition, in a previous study, the expression profiles of CXCL12/CXCR4/CXCR7 and CXCL13/CXCR5 in the positive and negative LNs of breast cancer patients were found. Based on the results of real-time quantitative reverse transcription PCR, the mRNAs for CXCL13 and CXCR4 were expressed at higher levels in LN ^+^ patients.^171^ Furthermore, vascular invasion was significantly associated with CXCL13 and CXCL12 expression in LN^+^ patients. CXCR5 expression was significantly higher in LN^+^ stage 3 patients than in stage 2 patients. Thus, CXCL12 and CXCL13/CXCR5 expression in the LNs of these breast cancer patients may be more relevant than other chemokines or receptors studied for LN involvement, resulting in a worse prognosis.^171^ Immune cells that express more chemokines, including CXCL13, may attract tumor cells and more immune cells with receptors to drain LNs. Several chemokines, such as CXCL13, have improved tumor cell survival (Fig. 6D).^172^

**Figure 7:**
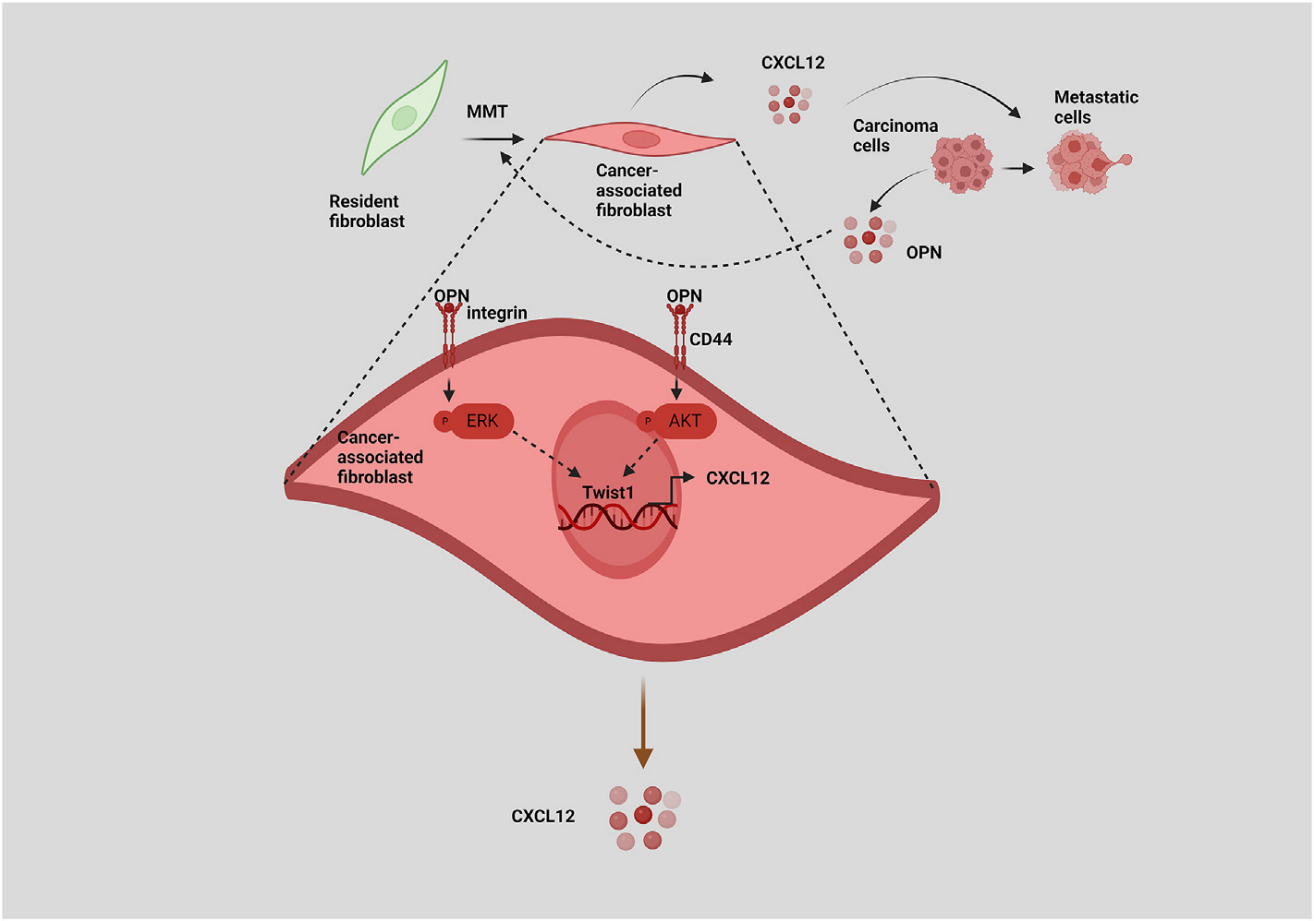
Osteopontin (OPN)-activated cancer-associated fibroblast (CAF)-derived C-X-C motif chemokine ligand 12 (CXCL12) promotes epithelial-to-mesenchymal transition (EMT) in breast cancer cells. OPN plays a critical role in the transdifferentiation of fibroblasts into CAFs via cluster of differentiation 44 (CD44) and integrin-mediated Ak strain transforming (Akt) and extracellular signal-regulated kinase (ERK)-dependent Twist1 expression and CXCL12, which eventually regulate breast cancer progression.

OPN, a chemokine-like protein, is vital in differentiating CAFs. In breast cancer, a transdifferentiation pathway for CAFs was recently proven to be regulated by tumor cell-derived OPN. OPN stimulates the expression of myofibroblastic genes, which in turn induces Twist1. CAFs provided via OPN secrete CXCL12. CXCL12 is an essential chemokine linked to cancer cell proliferation, inflammation, and immunological suppression. CXCL12, which is released by CAFs, causes EMT in cancer cells, increasing their migratory and angiogenetic potential (Fig. 7).^173^

## Role of ILs during obesity in menopausal women with breast cancer

Obesity raises the risk of breast cancer, especially in postmenopausal women.^174^^,^^175^ Breast cancer develops because of complex interactions between the environment and genetics that alter the immune and inflammatory systems in postmenopausal women, promoting carcinogenesis.^176^ Proinflammatory mediators that have recently been investigated in clinical and preclinical studies regarding obesity, postmenopausal, and breast cancer in women will also be examined as a vital part of this review.

### IL-1, IL-2, IL-6, IL-8, and IL-10

Roubert et al^177^ demonstrated a positive correlation between BMI and the secretion of IL-1, IL-6, and TNF-α, which promote breast cancer by stimulating wingless/integrated (Wnt) signaling in mammary tissue. Furthermore, Dias et al examined several inflammatory biomarkers, including oxidized low-density lipoprotein (ox-LDL), IL-1β, IL-6, TNF-α, white blood cells, neutrophils, and lymphocytes, in the nested case–control study. They discovered a strong correlation between ox-LDL, IL-1β, and TNF-α and post-menopausal breast cancer. TNF-α and ox-LDL variables exhibited inverse associations. On the other hand, IL-1β was associated with an increased risk of breast cancer. The findings suggested that inflammatory mediators could impact the development and progression of cancer.^178^ Furthermore, IL-1β genetic polymorphisms and TNBC susceptibility and breast cancer indicate the diagnostic and prognostic potential of these variants in postmenopausal individuals with breast cancer.^179^ This showed that the role of modifying factors and the illness phenotype must be taken into consideration when addressing the connection between IL-1β polymorphic variations and breast cancer risk.

In the study by Qiao and others, there is an interaction between menopause and BMI and IL-18 (IL-1 family member)–607 G/T genotype in the group of breast cancer patients with LN metastases and the non-metastasis group. For postmenopausal individuals who are overweight or obese, the IL-18–607 G/T genotype may raise the risk of LN metastases.^180^ This implies that endocrine, environmental, and genetic factors may contribute to the development of breast cancer. Furthermore, reduced objective response rates following neoadjuvant treatment in individuals with metabolic syndrome-related breast cancer are linked to elevated serum levels of adipocytokines and IL-18bp (binding proteins to IL-18 and inhibiting its function).^181^

A previous study discovered a correlation between a lower insulin level and the response to chemotherapy in patients with postmenopausal breast cancer. Additionally, researchers reported that before chemotherapy, the area under the curve of insulin was influenced by the BMI and basal levels of IL-1 and IL-8, while after chemotherapy, the main factors that predicted it were the BMI, basal levels of IL-1, and IL-8. ^182^ These findings suggested that the impact of chemotherapy on liver metabolism would be a feasible alternative explanation for the mechanism behind these changes. Furthermore, in a study involving women with early-stage breast cancer, fatigue was associated with interleukin 1 receptor antagonist (IL-RA) only after chemotherapy was administered; serum IL-12 was the sole variable found to be correlated with fatigue both before and following chemotherapy.^183^ Thus, these results indicated that chemotherapy could considerably influence the levels of circulating cytokines in post-menopausal women with breast cancer.

Recent research indicates that NK cells exposed to abnormal concentrations of adipokines, including IL-6, develop a dysfunctional phenotype and impaired functions.^184^ In a mouse model of postmenopausal breast cancer induced by diet-induced obesity, the expression of activating NK cell receptors, such as NKp46, NKG2D, and NKp30, is impaired.^184^

Iyengar et al recently showed in a clinical investigation (NCT00000611) that relatively high body fat levels were linked to an increased risk of invasive breast cancer and increased levels of circulating inflammatory factors, including IL-6, in postmenopausal women with a normal BMI.^174^ Remarkably, postmenopausal women tend to exhibit worse clinical outcomes even with appropriate BMI levels due primarily to increased fat volume.^174^ These results indicated that postmenopausal women's typical BMI categorization could not be a reliable indicator of their risk of developing breast cancer.

Studies have documented that omentin-1 inhibits the NF-κB signaling pathway, interferes with Toll-like receptor-4 (TLR4), and reduces the production of chemokines and cytokines such as TNF-α and IL-6, exhibiting anti-inflammatory characteristics.^185^ Christodoulatos et al^186^ discovered an independent negative correlation between plasma omentin-1 levels and breast cancer incidence in a postmenopausal cohort and inflammatory markers (TNF-α, IL-6). In addition, Laforest et al^73^ discovered that mammary adipocyte size is positively connected with tumor, metastatic stage, and tumor grade. Furthermore, they demonstrated a positive correlation between the diameter of breast fat cells and the expression of TNF and IL-6. In postmenopausal women, obesity-triggered cytokines contribute to inflammation and fat development; however, detailed research on the precise underlying mechanisms is inadequate.

A study by Madeddu et al^187^ on 216 individuals with breast cancer provided additional evidence for the involvement of peripheral inflammation and BMI in ER^+^ breast cancers. In this investigation, ER^+^ patients' IL-6 levels were considerably more significant than those of ER^−^ patients. Based on multivariate regression analysis, BMI, IL-6, and ROS predicted the tumor size, LN stage, and metastatic status of ER^+^ patients.^187^ These results suggest that an imbalance in adipokine production caused by obesity may create a pro-inflammatory milieu that promotes tumor growth, progression, and metastasis in ER^+^ breast cancer. Furthermore, recent research in ER^+^ BC revealed a correlation between high BMI and inflammation in white adipose tissue and aromatase expression and activity.^188^^,^^189^ Similarly, in postmenopausal women, there is a substantial correlation between elevated levels of aromatase and adipocyte size and indicators of subclinical systemic inflammation (IL-6),^189^ which promotes ER^+^ breast cancer cell survival and malignancy.

**Figure 8:**
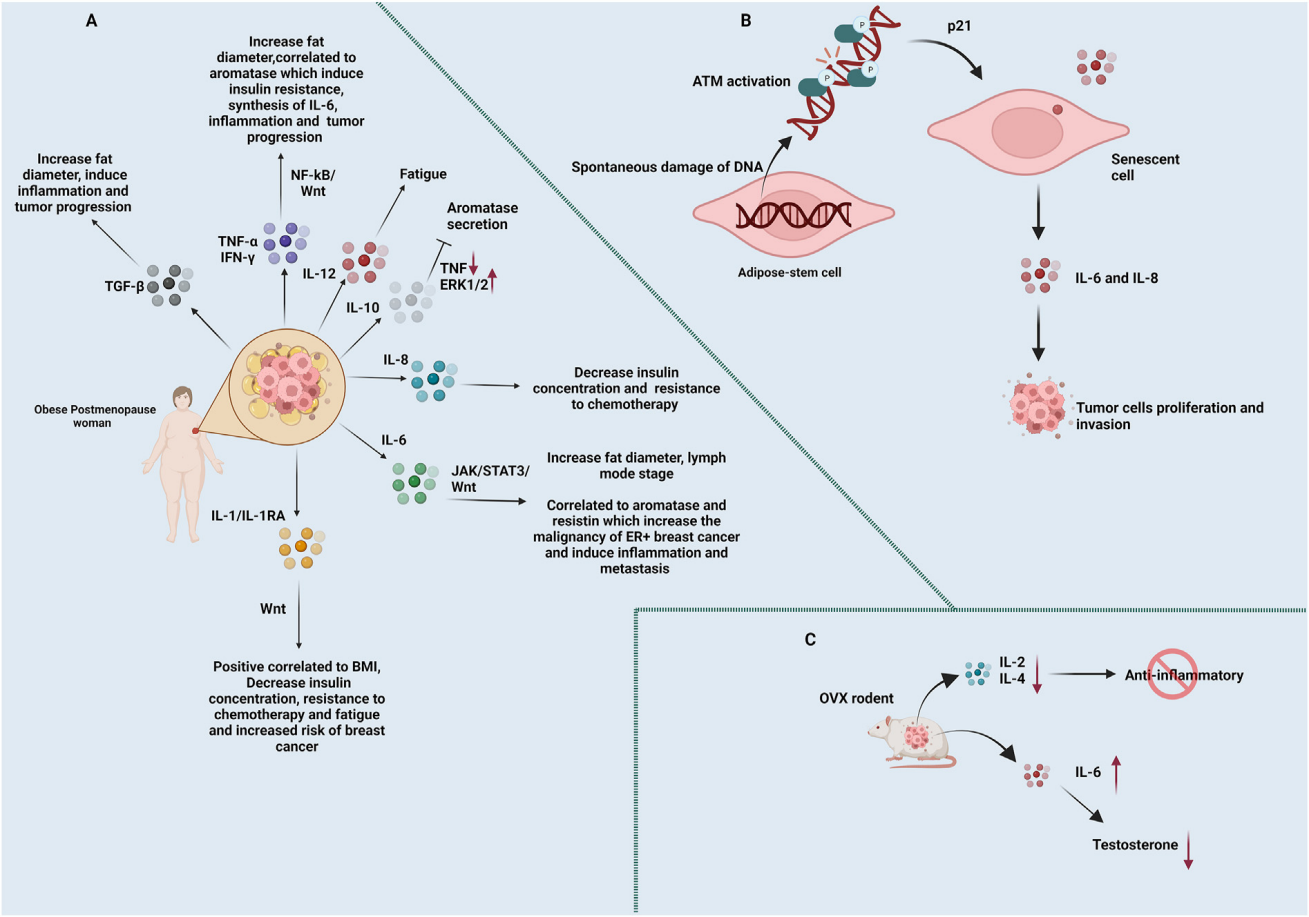
Obese and postmenopausal breast cancer women exhibit the involvement of pro-inflammatory cytokines (interleukins/ILs), as shown in the illustration. **(A)** Obese and postmenopausal women secrete a large amount of IL-1/IL-RA, which is correlated with body mass index (BMI). This secretion occurs via the Wnt signaling pathway in mammary tissue, promoting breast cancer. IL-1 also plays a role in decreasing insulin concentration, leading to chemotherapy resistance, fatigue, and increased risk of breast cancer. IL-6 is found to be correlated with BMI and can induce inflammation through the Janus kinase (JAK)/signal transducer and activator of transcription 3 (STAT3)/Wnt pathways. It is also correlated with aromatase and resistin, which are known to increase the malignancy of ER^+^ breast cancer and metastasis. Furthermore, IL-6 can impair the receptors of natural killer (NK) cells. The secretion of IL-8/C-X-C motif chemokine ligand 8 (CXCL8) induces insulin degradation and chemotherapy resistance. IL-10 secretion inhibits the secretion of aromatase via suppressing tumor necrosis factor (TNF)-stimulated extracellular signal-regulated kinase (ERK) 1/2 activation. In addition, the excessive secretion of IL-12 can cause fatigue. The secretion of interferon-gamma (IFN-γ) can induce inflammation via nuclear factor-kappa B (NF-kB). TNF-α, on the other hand, promotes breast cancer progression through the Wnt pathway. Additionally, TNF-α plays a role in increasing fat diameter and induces insulin resistance through aromatase while also being involved in the synthesis of IL-6. The secretion of transforming growth factor-beta (TGF-β) also contributes to an increase in fat diameter, induces inflammation, and promotes tumor progression. **(B)** The mechanism of how breast cancer susceptibility gene 1 (BRCA1)-deficient adipose-derived stem cells promote breast cancer progression. BRCA1-deficient adipose-derived stem cells are unable to repair both spontaneous and stress-induced DNA damage. This accumulation of DNA damage leads to a more persistently active ataxia-telangiectasia mutated (ATM) complex, which activates p21 and induces cellular senescence. In the senescent state, BRCA1-deficient adipose-derived stem cells secrete an increased number of inflammatory cytokines, which promotes breast tumor proliferation and invasion. **(C)** Ovariectomized (OVX) mouse models, commonly used in menopausal and breast cancer research, exhibit altered immunological parameters. In particular, the cytokine IL-6 up-regulates sensitized breast cancer cells to low levels of testosterone; on the other hand, the OVX model reduces the anti-inflammatory cytokines IL-2 and IL-4. The red color up arrow symbol (↑) represents up-regulation, while the down arrow symbol (↓) represents down-regulation.

Homologous recombination fails in BRCA1-deficient cancers, so their genomes have random mutations that can turn on oncogenes or turn off tumor suppressors, making them more aggressive. BRCA1-deficient cancers may develop and advance due to microenvironmental changes. BRCA1 mutations cause invasiveness in adipose stem cells.^190^ DNA damage accumulates due to defective DNA repair pathways in BRCA1-mutant adipose stem cells, which trigger the DNA damage response and the ataxia telangiectasia-mutated (AMT) pathway. Increased amounts of CDKN1A (p21), driven by ataxia-telangiectasia mutated (ATM) activation in cells, cause senescence and the release of inflammatory cytokines, including IL-6 and IL-8, which stimulate the growth and invasion of breast tumor cells (Fig. 8B).^190^ An analysis of the extent to which germline mutations impact cancer growth, invasion, and clinical prognosis is necessary and suggests additional research.

An *in vivo* investigation revealed that hyperglycemia may affect the microenvironment of breast cancer tumors in addition to its effects on metabolism. Co-culturing adipocytes with mesenchymal stromal/stem cells obtained from mammary adipose tissue increased the production of inflammatory cytokines or senescent phenotypes (IL-6, IL-8). They caused the xenografted breast cancer cells to exhibit a more aggressive and invasive phenotype.^191^ Based on these findings, it appears that glucose plays a role in the communication between breast mesenchymal stem cells and breast cancer cells, resulting in the development of a mesenchymal stem cell CAF-like phenotype characteristic of breast cancer cell stem cells.

Aromatase gene expression and increased estrogen levels are linked to white adipose tissue inflammation, and in obese postmenopausal women, these two variables together represent a substantial risk factor for breast cancer. A research study found that IL-10 suppresses TNF-stimulated ERK1/2 activation, which in turn decreases the expression of the aromatase gene in mesenchymal stem cells and adipose-derived stem cells.^192^ In addition, the Jagged1-Notch pathway was triggered in aromatase inhibitor-resistant breast cancer cells, resulting in macrophage differentiation toward M2 TAMs and increased production of IL-10. Consequently, M2 TAMs assisted in the development of drug resistance and metastasis.^193^ A recent study found that serum levels of cytokines, such as TNF-α, IL-6, IL-8, and IL-10, differ significantly between patients with breast cancer menopause and controls. These cytokines may be useful as prognostic and therapeutic markers.^194^

Human estrogen receptor-positive breast cancer cells, MCF-7 (luminal A subtype), were cultured in a rolling equipment system with human bone tissue from healthy or osteoporosis (OP) patients to create a humanized three-dimensional bone metastasis model. The work was conducted in a hypoxic environment.^195^ The study found that OP bone fragments increased the release of pro-inflammatory cytokines, such as TNF-α, IL-6, IL-1β, and IL-8, which regulate immune cell activity in the tumor microenvironment. IL-10, an anti-inflammatory cytokine that regulates other cytokines and immune responses, was also modulated. OP bone cultivated with breast cancer cells had higher IL-10 levels than healthy bone, indicating OP inhibits immune response. The decrease in osteoprotegerin (OPG)/receptor activator for nuclear factor KB ligand (RANKL) ratio, which favors bone resorption, further supports the idea that the OP microenvironment is more favorable to cancer cell bone metastasis than healthy ones.^195^ This finding demonstrated that in metastatic breast cancer, an altered bone microenvironment can exacerbate bone loss. The authors also found that OP bone cultures with breast cancer cells expressed more vascular endothelial growth factor (VEGF), VEGF-(receptor) R1, and VEGF-R2 than cell cultures with healthy cells, implying that these factors are even more closely associated with metastasis progression and invasion. Histological and immunohistochemistry analyses supported all these findings, revealing that three-dimensional culturing of OP bone with breast cancer cells led to enhanced local trabecular bone architectural change and an increase in cytokeratin 8/18 (CK8/18) positive breast cancer cells compared with healthy bone. This study highlighted and identified the significant distinctions in tumor growth and colonization between healthy and OP conditions.^195^ These three-dimensional models will be crucial for the creation of new advanced cell systems and for investigating the physiological processes involved in the interactions between cancer cells and their surroundings in the bone.

Furthermore, ovarian hormone deficiency in OVX animals leads to increased adiposity and enhanced aromatase synthesis in breast adipose tissue, attributed to a reduction in local IL-10 levels. Likewise, Alhallak et al^196^ found higher levels of IL-10 in the mammary fat tissue of obese premenopausal women, as well as higher expression of tumor inhibitory markers in breast tissue when compared with normal-weight women. The *in vitro* results showed that IL-10 administration promoted apoptosis and reduced cell proliferation in the mammary epithelial cell lines MCF10A and human mammary epithelial cells. However, there is currently insufficient evidence to support IL-10 as a potential preventive mediator against breast cancer; therefore, further research is required.

Menopause is challenging to study in women due to its onset and inter-individual variation. Pre-clinical models, such as OVX, have allowed researchers to examine the impact of loss of ovarian function on breast cancer.^197^ Obesity has little effect on mammary tumor incidence in rodents before OVX, but after OVX, obesity leads to more tumors and reduced anti-inflammatory cytokines (IL-2, IL-4).

Notably, in a rat model of obesity-associated postmenopausal mammary carcinoma, tumors that advanced after OVX exhibited a greater level of nuclear AR than tumors that regressed. Enzalutamide administration prevented the progression of tumors in rodents following OVX and prevented the formation of new tumors.^198^ Elevated levels of IL-6 in the plasma of obese rodents compared with lean rats sensitized breast cancer cells to reduced testosterone levels (Fig. 8C),^198^ demonstrating how cytokines and growth factors associated with obesity can influence the response of breast cancer tumors to steroid hormones and hormonal therapy, regardless of subtypes.

### TNF and TGF

A recent study revealed that activation or increases in multiple pathways, such as the TGF-β signaling pathway, the inflammatory response, the IL6-JAK-STAT3 and TNF-γ–NF–kB signaling, and the IFN-γ response, have been connected to the onset or progression of breast cancer.^199^ Further, postmenopausal gene expression and pathway changes were similar, supporting the association between menopause and weight increase and white adipose tissue inflammation. Interestingly, obesity and white adipose tissue inflammation affect blood biomarkers linked to breast cancer etiology, which correlated with breast molecular changes.^199^

Furthermore, Nascimento et al discovered that TNF-α controlled the synthesis of IL-6.^200^ In the adipose tissue of postmenopausal women with breast cancer, TNF-α increased the expression of aromatase, which in turn boosted estrogen.^201^ Consequently, obesity raised levels of TNF-α in the circulatory system, which raised the risk of breast cancer associated with insulin resistance and IL-6 production. A post-diagnosis case–control study has reported that higher circulating resistin is a risk factor for postmenopausal breast cancer, which correlates with inflammatory cytokines such as TNF-α and IL-6 ^202^. In this instance, altered resistin secretion as a component of obesity-induced chronic inflammation could promote the growth and advancement of breast tumors by causing an inflammatory fat microenvironment. In the future, clarifying the mechanism of increased resistin in breast cancer will be necessary. By reducing the inflammatory milieu in the breast epithelium, targeting resistin inhibition may be a sound therapeutic strategy to go beyond the resistance of the commonly used therapy methods for breast cancer. Fig. 8A displays the proinflammatory cytokines and pathways that contribute to the development of breast cancer in elderly, obese, and postmenopausal patients.

## Prospective theranostic therapies regarding cytokines, CXCs/CXCRs

As discussed above, aged breast cancer patients can produce many inflammatory ILs and CXCs and express various CXC receptors, which play a broad role in the occurrence and progression of breast cancer in aged patients. With the study of the molecular mechanism of aged breast cancer, cytokines are expected to become a new therapy for aged patients.^203^ Due to breast cancer cells' ability to secrete inflammatory ILs and CXCs in the tumor microenvironment, these ILs and CXCs may act as therapeutic targets in aged breast cancer patients.^84^^,^^94^^,^^117^ Several drug studies targeting ILs and CXCs/CXCRs in aged breast cancer patients are being performed, which may help strategize new treatment options.

Besides, several studies have clarified the potential significance of chemotherapy in focusing on pro-inflammatory cytokines and CXCs in breast cancer with aging.^204^ For instance, on March 31, 2023, a clinical trial addressing CXCR2 was found in the PubMed database (NCT01861054). Here is the link to the article: https://pubmed.ncbi.nlm.nih.gov/.^205^ In this trial, reparixin, a CXCR1/CXCR2 antagonist, was administered to patients with HER2-negative breast cancer before surgical tumor removal. The trial's treatment was well-tolerated and safe. Reparixin decreased the number of breast cancer stem cells in some patients.^205^ CXCR2 is also the receptor for CXCL/2/3/5/6/7/8, which provides a clue for researchers to target their receptors and evaluate the expression level of those ligands. In a randomized controlled trial (KCT0000939), desflurane was found to be beneficial to the immune response by maintaining the IL-2/IL-4 and CD4^+^/CD8^+^ T-cell ratios in breast cancer patients, providing an immune protective response.^206^ Yenidogan et al^207^ conducted a randomized trial and found that elderly breast cancer patients who received β-glucan treatment had significantly lower levels of TNF-α and IL-6. This suggests that β-glucan has anti-inflammatory properties for these patients.

According to a recent meta-analysis, physical activity is an essential element of a healthier lifestyle, specifically for obese women.^208^ A 2018 study found that obese postmenopausal breast cancer survivors can reduce inflammation (IL-6 and TNF-α) through a combination of resistance and aerobic exercise training.^209^ Furthermore, seven obese postmenopausal women participated in a 12-week diet and activity intervention as part of a pilot trial. The trial observed lower serum IL-6 levels compared with baseline.^210^ Furthermore, the 16-week therapeutic yoga program (TYP) randomized trial (NCT01654289) modifies the cytokine profile of IL-1β and IL-1Ra, which are considerably down-regulated in older women with heterogeneous cancer survival as well as decrease in overweight or obese cancer survivors.^211^ The study found that TYP led to a significant reduction in the levels of cytokines associated with chronic inflammation in a heterogeneous group of cancer survivors. These results suggest that regular physical activity can help reduce inflammation and obesity. However, while the findings are promising, further well-designed and well-described controlled clinical studies are needed to confirm the effects of physical activity and understand the underlying mechanisms.

To treat breast cancer, anti-CXCL12 aptamers, CXCR4 antagonists, and anti-CXCR4 monoclonal antibodies have been developed and tested in clinical studies. For instance, Spexis created the cyclic synthetic peptide balixafortide (POL6326), which consists of 14 amino acids. It is a very effective and particular chemokine receptor CXCR4 antagonist that suppresses tumor development and proliferation while also boosting the immune system within the microenvironment of breast tumors.^212^ In addition, in patients with pretreated, relapsed metastatic HER2-negative breast cancer, the new CXCR4 antagonist balixafortide (POL6326, NCT01837095, phase 1) was evaluated in combination with eribulin.^213^ After evaluating patients, the initial anti-cancer activity seems beneficial. The objective response rate was 30% (16/54), and a therapeutic benefit was noted in 44% (24/54) of the patients. This combination of balixafortide and eribulin demonstrated good tolerability and safety in the dose-escalation assessment, comparable to either drug used alone.

There is evidence that flavonoid apigenin in fruits and vegetables can target cellular pathways such as IRAK1/4, p38-MAPK, and NF-kB. It is crucial for clearing senescent cells and reducing SASP.^204^ It has been suggested that apigenin is a promising adjuvant treatment for cancers.

A study on elderly individuals with early-stage breast cancer and chemotherapy reported high levels of IL-12, IL-17, MIP-1, and G-CSF before treatment. These levels fluctuated after breast cancer treatment and dropped considerably two years later. This observational study found a connection between proinflammatory cytokines such as IL-1β and IL-6 and chemotherapy-related cognitive impairment.^214^ Furthermore, recently, chemotherapy patients performed better on subjective cognitive perception tests. The postchemotherapy subgroups with lower semantic verbal fluency had increased IL-13 levels. IL-10 was associated with better cognitive abilities in the prechemotherapy and control groups, while IL-5 and IL-13 were associated with inferior cognitive abilities. These mediation analysis results suggest that cancer status may affect verbal proficiency regardless of anxiety.^215^ It is necessary to conduct more extensive prospective trials to confirm these correlations, even though researchers are still studying the precise mechanisms of neurotoxicity. Before confirming these correlations, more extensive prospective trials must regularly employ biomarkers for risk-stratifying individuals.

In the study by Brouwers et al,^216^ chemotherapy had an insignificant effect on the expression levels of aging biomarkers such as IL-10, TNF-α, IGF-1, MCP-1, and IL-6 in breast cancer patients. Although these biological indicators do change during and after chemotherapy, there is no substantial evidence to support an acceleration of the aging process that is clinically relevant. It is a significant finding since it highlights that elderly breast cancer patients should not be denied chemotherapy only because of their advanced age.

Additionally, another study investigated variations in the *ex vivo* expression of inflammatory mediators by immune cells in breast cancer patients and normal controls following stimulation.^217^ From pre-treatment to six months post-treatment and throughout the following year, breast cancer patients in this study had considerably more significant increases in stimulated cytokine production than controls; these effects were most prominent in women who received surgery, chemotherapy, and radiotherapy.^217^ The same results were found by Bower et al.^218^ In this experiment, women who received multimodal therapy observed significant increases in comorbidities, highlighting the clinical significance of chronic inflammation.

A study report revealed that activated T cells significantly increased IFN-γ and IL-2/9/10/22/23 levels, which could be reversed by administering Rg3-CNT. *In vivo*, Rg3-CNT reduced the development of TNBC cells by blocking the PD-1/PD-L1 axis. Rg3-CNT enhanced Rg3's anti-cancer activity in TNBC. These discoveries shed new light on the process by which Rg3-CNT inhibits the growth of TNBC. Rg3-CNT could be a viable treatment approach for TNBC immunotherapy.^219^

Table 3 lists clinical and preclinical trials aiming to target inflammatory cytokines and CXCs/CXCRs. These targeting strategies have shown potential in treating breast cancer, especially in older patients. Thus, developing drugs targeting ILs/CXCs/CXCRs can help treat breast cancer, where immune inflammation plays a vital role.

**Table 3:** Clinical and preclinical trials targeting pro-inflammatory cytokines and CXC chemokines in elderly patients with breast cancer.

Inhibitor/Drug/Physical activities	Target	Study type	Finding/Outcome	Trial phase	Trial number
Atezolizumab plus Nab-Paclitaxel	CXCL10	Clinical	Atezolizumab treatment does not prevent CXCL10 levels^232^	Phase 1b	NCT01633970
Bevacizumab chemotherapy	IL-8	Clinical	Low plasma IL-8 levels during chemotherapy (Bevacizumab) are predictive of excellent long-term survival in metastatic breast cancer^233^	II	NCT00979641
Docetaxel or vinorelbine	CXCL13	Clinical	High CXCL13 was associated with favorable distant disease-free survival (DDFS), especially in the TNBC^234^		FinHer trial (ISRCTN76560285)
Fecal *Akkermansia muciniphila* (AM)	IL-6	Clinical	AM was inversely associated with the inflammatory cytokine IL-6, which suggests a lesser role for AM in mitigating systemic inflammation^235^	0-II	NCT02224807
GeparNuevo neoadjuvant trial	CXCL10	Clinical	CXCL10 gene linked to antigen presentation and IFN signaling was strongly correlated with pathologic complete response in durvalumab but not placebo^236^	II	NCT02685059
Lysosomotropic drugs	CXCL5, CXCL8	Clinical	Because autophagy is inhibited as a result of lysosomal activity, ROS are produced, which trigger inflammatory genes (CXCL5, CXCL8)^237^	I-III	NCT00943839
Lapatinib	IL-8	Clinical	Lapatinib treatment down-regulation the IL-8 level in the TME of LABC patients^238^	I	BMS-986253
Nature-based walking	TNF-γ, IL-1ß, IL-6, TGF-ß, IL-10, IL-13	Clinical	Physical activity (PA) effects on pro and anti-inflammatory cytokines such as TNF-γ, IL-1ß, IL-6, TGF-ß, IL-10, and IL-13^239^	I-III	NCT04896580
Propofol and desflurane	IL-2/IL-4 and CD4+ /CD8+ T cell ratio	Clinical	Propofol and desflurane surgery improve the immune system, including IL-2/IL-4 and CD4+/CD8+ T cell ratios, during the perioperative period^206^		KCT0000939
Reparixin	CXCR1	Clinical	Reparixin was well-tolerated. In several patients, flow cytometry results showed fewer cancer stem cells, suggesting CXCR1 targeting^205^	Phase Ib	NCT01861054
Therapeutic yoga (TYP)	IL-1b/IL-1ra/IL-6/IL-8/IL-10/IFN-γ/TNF-α	Clinical	In a group of cancer survivors, TYP significantly reduced the levels of circulating cytokines linked to chronic inflammation^211^		NCT01654289
High-Intensity interval training (HIIT)	IL-4/IL-6/IL-10/TNF-α	Clinical	The implementation of HIIT seems to be effective in reducing the plasma and serum levels of various pro-inflammatory markers (TNF-α, IL-6), and in increasing the expression of iIL-4 and IL-10^240^		
Practice-based opportunities for weight reduction (POWER)	IL-1α, IL6, and IL8	Clinical	Did not have favorable improvements in inflammatory cytokines^241^		NCT01871116
18-month behavioral weight-loss trial	IL-6	Clinical	Weight loss has a significant effect on the change in IL-6 level^242^		NCT01441011
Exercise	IL-6	Clinical	Exercise was found to have a more beneficial effect on IL-6 levels due to altering body fat and fitness^243^		NCT01511276
Aerobic (walking) + resistance exercise	IL-10, serum IL-6, IL-8, TNFα, IL-6/IL-10 ratio, IL-8/IL-10 ratio, TNFα/IL-10 ratio	Clinical	Reduces IL-10 and serum IL-6, IL-8, TNFα, IL-6/IL-10 ratio, IL-8/IL-10 ratio, TNFα/IL-10 ratio no effect^244^		NCT01147367
Capsules of synbiotic supplements	TNF-α	Clinical	8-week synbiotic consumption by overweight and obese postmenopausal breast cancer patients had beneficial effects on TNF-α^245^		IRCT20091114002709N49
Balixafortide plus eribulin	CXCR4	Clinical	Polyphor CXCR4 antagonist balixafortide, in combination with the chemotherapeutic drug eribulin, was studied in the phase 1 trial for women with HER2-negative metastatic breast cancer, which resulted in a 30% objective response rate^213^	I	NCT01837095
Vitamin D3	TNF-α	Clinical	Decreased in inflammatory biomarker (TNFα) after supplemented vitamin D3 in breast Cancer^246^	I-III	IRCT2017091736244N1
16 weeks of exercise	IL-8/CXCR2	Clinical	16 weeks of exercise training change BCS resting IL-8 and CXCR2 levels^247^	I-III	NCT03760536
Administration of IL-2	IL-2	Clinical	Cyclic administration of IL-2 considerably increases the survival time of postmenopausal patients with endocrine-dependent metastatic breast cancer^101^^,^^103^		
β-Glucan	IL-6 and TNF-α	Clinical	The administration of β-glucan plays a role in the downregulation of these cytokines^207^		
Antroquinonol (AQ) and 4-Acetylantroquinonol (AAQB)	IL-10 and TNF-α	Preclinical	AQ and 4-AAQB inhibited these inflammatory cytokines in MCF-7 breast cancer cells^248^		
chemotherapy	IL-5/10/13	Clinical	Higher levels of cytokines IL-5 and IL-13 were significantly associated with lower subjective cognitive complaints, and higher IL-10, on the other hand, was associated with better subjective perceived cognition^215^		
Pain palliative therapy	IL-2/6/TNF-α	Clinical	Responders to therapy had higher IL-2 and lower IL-6/TNF- concentrations^100^		
Docetaxel and Cyclophosphamide	Il-6/10 and TNF-α	Clinical	IL-6 was unchanged, and IL-10 and TNF-α suggested a minor biological aging effect of chemotherapy^216^		
Conditioned medium (CM) from senescent *Spalax*	IL-α	Preclinical	Under the impact of *Spalax* CM, IL-1α dysregulation, particularly a reduction in membrane-bound IL-1α, is crucial for decreasing inflammatory production in cancer cells, which in turn inhibits cancer cell motility^249^		
Death receptors (DR) 5	TNF-α and TRAIL	Preclinical	DR5 selective TRAIL variant (DHER) enhances apoptosis of senescent cancer cells^129^		
Ginsenoside Rh2	IL-1/IL-6/IL-8	Preclinical	Rh2 significantly reduced the mRNA level of CXCL1, IL-6 and IL-8^107^^,^^250^		
Ginsenoside Rg3	IFN-γ/IL-2/IL-9/IL-10/IL-22/IL-23	Pre-clinical	Rg3-CNT was able to reduce IL-2, IL-9,IL-10,IL-22, and IL-23^219^		
IL-22 neutralization antibody	IL-1β- and IL-23	Pre-clinical	Blocking the action of IL-22 may significantly reduce the development of breast cancer tumors caused by IL-1β and IL-23^251^		
Kallistatin	TGF-β	Preclinical	Kallistatin stops EMT by blocking the TGF-β caused miR-21-Akt–NF–B pathway and oxidative stress^252^		
Metformin	TGF-β	Preclinical	Metformin prevents TGF-induced EMT from cancer stem cells to the fibrosis induced by age^253^		
MDM2 inhibitors	IL-1α/IL-6	Pre-clinical	The inhibitors inhibit senescent cells from producing IL-6 and IL-1α, typical SASP factors. It was caused by genotoxic stress^254^		
Tacalcitol (PRI 2191)	TGF-β and IL-6	Pre-clinical	Young mice treated with PRI-2191 released more significant levels of IL-17A than untreated, control mice, and older OVX animals showed a reverse effect (decreased IL-17A secretion)^255^		
Quercetin derivative (QD3)	IL-1β and IL-8	Pre-clinical	The senolytic effect of QD3 was found to be associated with a reduction in the levels of IL-1β and IL-8 in etoposide-induced senescent MDA-MB-231 breast cancer cells^256^		
Tamoxifen	IL-18	Clinical	There was a significant reduction in serum levels of IL-18 after tamoxifen therapy^257^		
Consumption of specific antioxidants, including β-tocopherol, zinc, and selenium	IL-10	Clinical	This consumption may reduce breast tissue-level inflammation by decreasing oxidative stress and proinflammatory cytokine (IL-10) expression in postmenopausal women^258^		
Surgery alone or with chemotherapy, radiation, or both	sTNF-RII, IL-1RA, and IL-6	Clinical	Even treatment, sTNF-RII, IL-6, and IL-1RA correlate with worsened physical functioning, and sTNF-RII correlates with increased pain.^259^		

## Conclusions

We sought to clarify the expression, fundamental molecular processes, and significant functions of cytokines, CXCs, and CXCRs in breast cancer in older patients. Breast cancer development, metastasis, and progression are significantly influenced by certain moderators called CXCs and their corresponding receptors, which are found in aged and postmenopausal women with obesity. These moderators include IL-1, IL-6, IL-8, IL-10, IFN, TGF, and TNF, as well as CXCL1, CXCL8, CXCL9, CXCL10, CXCL11, CXCL12, and CXCL13. Breast cancer in older individuals is prevented from developing and progressing when cytokines, CXCs, and CXCRs are inhibited. This may help in the early detection and treatment of breast cancer in elderly patients. Besides physical activity, it was found to have a more beneficial effect on obese postmenopausal patients. Surprisingly, IL-10 has demonstrated beneficial effects in both in *vivo* and in *vitro* obese postmenopausal breast cancer models. However, researchers have conducted only a limited number of investigations on IL-10. There is currently inadequate evidence to suggest IL-10 as a possible preventative mediator in obese breast cancer patients against an enzyme (aromatase). Further research is necessary to evaluate its efficacy.

## Future prospectives

Insufficient knowledge regarding the complex relationship between cytokines, CXCs, and breast cancer in older women may hinder the development of therapeutic agents. Most cytokines and CXCs are highly expressed in breast cancer in elderly and obese postmenopausal patients. In contrast, they are also involved in the pathogenesis of breast cancer in older women by causing inflammation. However, their molecular bases have not yet been fully explored. Therefore, more investigations are needed to improve the understanding of the role of ILs and other CXCs in elderly breast cancer postmenopausal women, which may help further develop therapeutic agents for this respective breast cancer population. These investigations will not only be of scientific interest. However, they could also prove helpful for breast cancer treatment in elderly patients.

## CRediT authorship contribution statement

**Amin Ullah:** Formal analysis, Methodology, Visualization, Writing – original draft, Writing – review & editing. **Rajeev K. Singla:** Software, Writing – review & editing. **Dan Cao:** Formal analysis, Writing – review & editing. **Boyang Chen:** Formal analysis, Software. **Bairong Shen:** Conceptualization, Formal analysis, Funding acquisition, Investigation, Methodology, Project administration, Resources, Supervision, Writing – review & editing.

## Funding

This work was supported by the 10.13039/501100001809National Natural Science Foundation of China (No. 32070671, 32270690), the COVID-19 research projects of 10.13039/501100013365West China Hospital 10.13039/501100004912Sichuan University (China) (No. HX-2019-nCoV-057), and the regional innovation cooperation between Sichuan and Guangxi Provinces, China (No. 2020YFQ0019).

## Conflict of interests

The authors say they have no commercial or financial ties that could be seen as a conflict of interest in our study. Because of this, there were no conflicts of interest in the study.
